# Significance of the geriatric nutritional risk index and body composition as prognostic indicators in gastric cancer patients

**DOI:** 10.3389/fonc.2025.1719952

**Published:** 2026-01-12

**Authors:** Wenzhi Wu, Wenchang Yang, Jingnong Liu, Yuandi Wang, Zhengzhao Wang, Xianxiang Zhang

**Affiliations:** 1 Department of Gastrointestinal Surgery, The Affiliated Hospital of Qingdao University, Qingdao, Shandong, China; 2 Qingdao Medical College, Qingdao University, Qingdao, Shandong, China

**Keywords:** body composition, gastric cancer, geriatric nutritional risk index, nutrition, survival

## Abstract

**Background:**

A poor prognosis in patients with gastric cancer (GC) is independently linked to malnutrition and CT-defined low muscle mass. However, the combined effects on prognosis outcomes are not fully elucidated. This study systematically evaluated the synergistic effects of body composition parameters and nutritional indicators in predicting the prognosis of gastric cancer.

**Methods:**

This retrospective study included 986 middle-aged and elderly patients with stage II/III GC who underwent surgical resection. Body composition parameters, including the skeletal muscle index (SMI), skeletal muscle density (SMD), and various adipose tissue indices, were evaluated by a single cross-sectional computed tomography image at the L3 level. The combined indices were defined as the product of the geriatric nutritional risk index (GNRI) and body composition parameters. Prognostic analyses were conducted using the Kaplan-Meier method.

**Results:**

The 986 patients were divided into a training cohort (n = 690) and a validation cohort (n = 296) at a 7:3 ratio. The median (interquartile) age was 71 (67-75) years, and 623 (63.2%) patients were male. In the training cohort, the median values of the SMI, SMD, adipose tissue parameters, and their combinations with the GNRI differed significantly by sex. Patients were classified into sex-specific quartiles (Q1-Q4) on the basis of SMI × GNRI score. Both overall survival (OS) and disease‐free survival (DFS) were significantly different across these quartiles (P < 0.001). Although all body composition parameters and their combinations with the GNRI were independent predictors of OS and DFS according to multivariate analysis, the combination of the SMI × GNRI demonstrated superior prognostic performance compared with other indices in the prediction of OS (c‐statistics: 0.749, AICc: 3842.9) and DFS (c‐statistics: 0.731, AICc: 4174.7). These results remained consistent across the stratified analyses. The validation cohort confirmed that the SMI × GNRI exhibited greater predictive and discriminative power than the other indices did.

**Conclusions:**

A robust and readily applicable tool for prognostic assessment, the SMI×GNRI index effectively predicts survival outcomes in middle-aged and elderly stage II/III GC patients. Further prospective studies are needed to validate its effectiveness across diverse populations and clinical settings.

## 1 Introduction

Gastric cancer (GC) is the fifth most common malignant tumor in the world and has high morbidity and mortality (1). For GC patients with the same tumor-node-metastasis (TNM) stage, there are also significant differences in prognosis between stage II/III and stage I (2–4). This heterogeneity emphasizes the need for more sophisticated prognostic tools to better reflect the individual characteristics of patients (3, 5). Especially in middle-aged and elderly patients with stage II/III, malnutrition and adverse changes in body composition often exist at the same time (6–9), which is closely related to poor survival (10), but it is not captured by the TNM stage system alone. It is urgent to determine combined nutritional indicators to develop individualized risk assessment and treatment strategies for middle-aged and elderly patients with stage II/III GC.

The geriatric nutritional risk index (GNRI), an objective nutritional screening tool derived from serum albumin and ideal body weight (7), is a well-established prognostic biomarker across a variety of cancers (7, 11–13). Its significant prognostic value has been extended to non-elderly individuals (14). Concurrently, the prognostic significance of computed tomography (CT)-defined body composition in cancer, which includes key parameters such as skeletal muscle (SM), visceral adipose tissue (VAT), and subcutaneous adipose tissue (SAT), is well-recognized (15). Among these, CT-defined low muscle mass, characterized by a degenerative loss of skeletal muscle, is linked to adverse clinical outcomes in cancers (16, 17). The GNRI and skeletal muscle index (SMI) are each considered important predictors, and they are biologically related (18–20). Malnutrition can directly lead to muscle loss, and low muscle mass may aggravate metabolic dysfunction (18–20). As confirmed by Wang et al., a synergistic model incorporating GNRI, CT-defined low muscle mass, and VAT provided a more accurate risk assessment for death and recurrence in elderly intrahepatic cholangiocarcinoma patients (21). In another study, Go et al. showed that their model consist of GNRI combined with CT-defined low muscle mass significantly optimized prediction efficiency in diffuse large B-cell lymphoma (22). However, the synergistic prognostic potential of these two indicators has not been fully explored in GC.

We assume that the combined index of SMI and GNRI can provide better prognostic stratification. Therefore, this study aims to systematically evaluate whether the composite index is superior to other body composition parameters and their combinations with GNRI to predict the survival of middle-aged and elderly patients with stage II/III GC.

## 2 Methods

### 2.1 Study population

The conduct of this study was in full compliance with the principles outlined in the Declaration of Helsinki, following approval from the hospital’s ethics committee (Approval No. QYFYWZLL30313). The requirement for informed consent was waived because of the retrospective nature of the study.

A total of 986 patients with stage II/III GC who received radical surgery (RS) at the Affiliated Hospital of Qingdao University between January 2018 and June 2022 were included (**Supplementary Figure 1**). Patients were randomly divided in a 7:3 ratio, with 70% (690) allocated to the training set for model development and the remaining 30% (296) allocated to the validation set for model validation. The inclusion criteria were as follows: (1) aged 50–80 years; (2) pathologically confirmed stage II/III GC according to the TNM Classification of Malignant Tumors, 8th Edition (23); (3) received RS; (4) blood testing was performed within 14 days prior to surgery; and (5) CT within 30 days prior to surgery. The exclusion criteria were as follows: (1) patients with a history of other cancers; (2) factors that may affect peripheral blood cell counts, including acute or chronic infections, diseases of the immune system, etc; (3) patients lacking clinical data; and (4) patients lost to follow-up.

### 2.2 Surgery and postoperative management

All enrolled patients received RS combined with systematic lymphadenectomy, with the method for digestive tract reconstruction decided by the operating surgeon. The postoperative complications within 30 days after surgery were assessed according to the Clavien-Dindo classification system for surgical complications (24). For patients with concurrent multiple complications, the grading was based on the severity of the most critical complication. Major POCs were defined as Grade II or higher. The standardized follow-up protocol, conducted over a 5-year period or until death (whichever came first), scheduled assessments at six-month intervals, comprising physical examinations, laboratory tests, and enhanced CT scans. The last follow-up date was July 31, 2025. The study’s primary endpoints were overall survival (OS) and disease-free survival (DFS). OS refers to the duration from surgery to either death or the final follow-up, while DFS measures the time from surgery to tumor recurrence or the last follow-up. Individualized adjuvant therapy was administered to all patients unless contraindications existed or consent was declined. Management upon recurrence was guided by evidence-based guidelines, the patient’s clinical status, and informed consent.

### 2.3 Measurements of body composition parameters

The segmentation of the different abdominal tissues was performed semiautomatically by the analysis software SliceOMatic V5.0 (Tomovision) based on characteristic radiodensity ranges (**Supplementary Figure 2**). The skeletal muscle area (SMA), subcutaneous adipose tissue (SAT) area (SATA) and visceral adipose tissue (VAT) area (VATA) were evaluated from a single image at the third lumbar vertebra (L3) using Hounsfield unit (HU) thresholds of −29 to +150 for SM, −190 to −30 for SAT, and −150 to −50 for VAT, respectively (9). The total adipose tissue area (TATA) was calculated as the sum of the SATA and VATA. Skeletal muscle density (SMD) was assessed by estimating the mean HU value of the SMA. To account for variations in patient height, each area was normalized by dividing by the square of the patient’s height (m^2^), yielding the skeletal muscle index (SMI), subcutaneous adipose tissue index (SATI), visceral adipose tissue index (VATI) and total adipose tissue index (TATI), respectively. The formulas for calculating these indices are as follows: SMI (cm^2^/m^2^) = SMA/height^2^, SATI (cm^2^/m^2^) = SATA/height^2^, VATI (cm^2^/m^2^) = VATA/height^2^, and TATI (cm^2^/m^2^) = TATA/height^2^.

### 2.4 Clinical data assessment

This study collected the following data from electronic medical records: demographic and clinical characteristics (age, sex, body mass index [BMI], hypertension, diabetes, and neoadjuvant/adjuvant therapy); laboratory parameters (neutrophil count, lymphocyte count, hemoglobin, cholesterol, low-density lipoprotein [LDL], high-density lipoprotein [HDL], albumin, carcinoembryonic antigen [CEA] and carbohydrate antigen 19–9 [CA19-9] levels); and pathological features (tumor location, maximal size, TNM stage, and tumor differentiation).

The preoperative GNRI score was calculated as [14.89×serum albumin level (g/dL) + 41.7× preoperative body weight (kg)/ideal body weight (kg)] (13). Ideal body weight was sex-specifically computed: for men, height (cm) - 100 - ([height (cm) - 150]/4); for women, height (cm) - 100 - ([height (cm) - 150]/2.5) (13). A ceiling value of 1 was applied to the body weight ratio when a patient’s preoperative weight exceeded their ideal weight. The combined nutritional indices were as follows: SMI×GNRI, SATI×GNRI, VATI×GNRI, TATI×GNRI, SMD×GNRI. When constructing the combined nutritional indices of GNRI and body composition parameters, we used the multiplicative Model (GNRI × body composition parameters). This approach is based on the following considerations: First, from the perspective of clinical pathophysiology, malnutrition (reflected by low GNRI) and abnormal body composition (such as muscle loss) may have a synergistic amplification effect on the prognosis of patients, rather than a simple superposition effect. The multiplicative model can better capture this nonlinear interaction (25). Secondly, statistically, the product term is used as an interaction term to formally test whether body composition has a moderating effect on the relationship between GNRI and research outcomes.

### 2.5 Statistical analysis

For continuous variables, the unpaired Student’s t test was used for those with a normal distribution (expressed as the mean ± standard deviation), the Wilcoxon rank sum test or Kruskal-Wallis test was applied for abnormal distribution data (expressed as median and interquartile range), and the Chi-square test or Fisher’s exact test was used for categorical variables (expressed as percentages). OS and DFS were estimated by the Kaplan-Meier method and compared between groups using the log-rank test. Potentially significant risk factors (P < 0.05) in the univariate analysis were included in the multivariate Cox regression analysis. In the multivariate Cox regression analysis in GC patients, we adjusted for the key clinical and pathological variables identified through univariate analysis that would affect the OS or DFS of GC patients. Evaluation of prognostic efficacy for the different indicators involved Harrell’s c-statistic and the corrected Akaike information criterion (AICc). The statistical analyses relied on SPSS v27.0 (IBM, Armonk, NY) and R version 4.2.2, with a two-sided P < 0.05 deemed statistically significant.

## 3 Results

### 3.1 Baseline characteristics

This study included 986 patients, distributed into a training cohort (n=690) and a validation cohort (n=296) (**Supplementary Table 1**).

**Figure 1:**
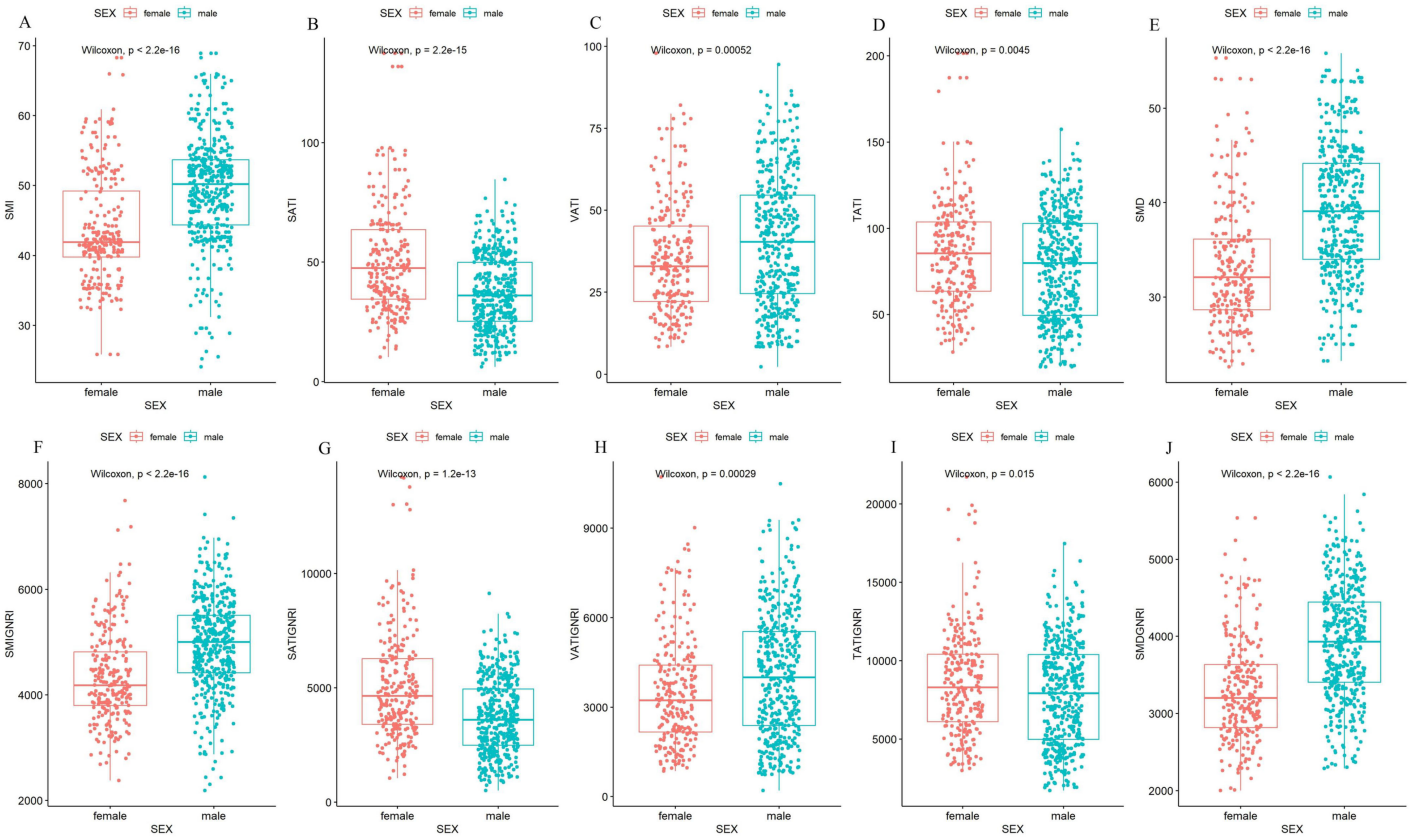
Comparison of body composition parameters and the combinations with GNRI according to sex. **(A)** Comparison of SMI by sex; **(B)** Comparison of SATI by sex; **(C)** Comparison of VATI by sex; **(D)** Comparison of TATI by sex; **(E)** Comparison of SMD by sex; **(F)** Comparison of SMI×GNRI by sex; **(G)** Comparison of SATI×GNRI by sex; **(H)** Comparison of VATI×GNRI by sex; **(I)** Comparison of TATI×GNRI by sex; **(J)** Comparison of SMD×GNRI by sex. Due to the abnormal distribution of the data, the data was compared using Wilcoxon rank sum test between two groups. SMI, skeletal muscle index; SATI, subcutaneous adipose tissue index; VATI, visceral adipose tissue index; TATI, total adipose tissue index; SMD, skeletal muscle density; GNRI, geriatric nutritional risk index.

In our cohort, the median patient age of 71 years and a male predominance (623 patients, 63.2%). The baseline characteristics of the training cohort are presented in **Supplementary Table 2**. A total of 139 patients had major POCs, including 92 (13.3%) in the training cohort and 47 (15.9%) in the validation cohort. The median values of the SMI, SATI, VATI, TATI and SMD differed significantly by sex (**Figure 1**, **Supplementary Table 2**). Similarly, notable differences were observed for SMI×GNRI, SATI×GNRI, VATI×GNRI, TATI×GNRI, and SMD×GNRI across sex-specific subgroups (**Figure 1**). Consequently, patients were stratified into sex-specific quartiles (Q1-Q4) on the basis of SMI×GNRI levels. The corresponding quartiles from the male and female subgroups were then combined, creating four new groups comprising 172, 173, 173, and 172 patients, respectively (**Table 1**).

**Table 1:** Patient characteristics according to sex-specific quartiles of SMI×GNRI levels.

Variables	Q1 (n=172)	Q2 (n=173)	Q3 (n=173)	Q4 (n=172)	P value
Age (years)	72.0 (68.0-76.0)	70.0 (66.0-75.0)	70.0 (67.0-74.0)	71.0 (64.0-74.0)	0.006*
Male, n (%)	110 (64.0%)	111 (64.2%)	111 (64.2%)	110 (64.0%)	1.000
BMI≥25(kg/m^2^), n (%)	34 (19.8%)	50 (28.9%)	64 (37.0%)	115 (66.9%)	<0.001*
Hypertension, n (%)	59 (34.3%)	67 (38.7%)	62 (35.8%)	56 (32.6%)	0.671
Diabetes, n (%)	53 (30.8%)	41 (23.7%)	42 (24.3%)	48 (27.9%)	0.402
NRS-2002≥3, n (%)	127 (73.8%)	115 (66.5%)	107 (61.8%)	92 (53.5%)	<0.001*
Major POCs, n (%)	24 (14.0%)	19 (11.9%)	26 (15.0%)	23 (13.4%)	0.726
CEA ≥5 (ng/ml), n (%)	54 (31.4%)	46 (26.6%)	35 (20.2%)	37 (21.5%)	0.065
CA19-9 ≥30 (U/L), n (%)	114 (66.3%)	110 (63.6%)	92 (53.2%)	88 (51.2%)	0.008*
Tumor location, n (%)					0.900
Lower and Mix	106 (61.6%)	109 (63.0%)	112 (64.7%)	112 (65.1%)	
Middle and Upper	66 (38.4%)	64 (37.0%)	61 (35.3%)	60 (34.9%)	
Tumor size, cm	3.5 (2.5-5.0)	3.5 (2.5-5.0)	3.5 (3.0-5.0)	3.9 (3.0-5.5)	0.511
Pathological T stage, n (%)					<0.001*
T1	3 (1.7%)	9 (5.2%)	7 (4.0%)	14 (8.1%)	
T2	28 (16.3%)	35 (20.2%)	56 (32.4%)	56 (32.6%)	
T3	52 (30.2%)	53 (30.6%)	32 (18.5%)	45 (26.2%)	
T4	89 (51.7%)	76 (43.9%)	78 (45.1%)	57 (33.1%)	
Pathological N stage, n (%)					<0.001*
N0	17 (9.9%)	27 (15.6%)	26 (15.0%)	23 (13.4%)	
N1	22 (12.8%)	42 (24.3%)	60 (34.7%)	53 (30.8%)	
N2	59 (34.3%)	57 (32.9%)	48 (27.7%)	52 (30.2%)	
N3	74 (43.0%)	47 (27.2%)	39 (22.5%)	44 (25.6%)	
Tumor differ- entiations, n (%)					0.600
Poorly	152 (88.4%)	153 (88.4%)	148 (85.5%)	147 (85.5%)	
Moderately	17 (9.9%)	17 (9.8%)	21 (12.1%)	17 (9.9%)	
Well	3 (1.7%)	3 (1.7%)	4 (2.3%)	8 (4.7%)	
Nerve invasion, n (%)	76 (44.2%)	77 (44.5%)	57 (32.9%)	68 (39.5%)	0.099
Vascular invasion, n (%)	94 (54.7%)	94 (54.3%)	99 (57.2%)	92 (53.5%)	0.909
Adjuvant therapy, n (%)	95 (55.2%)	113 (65.3%)	114 (65.9%)	119 (69.2%)	0.043*
White blood cell count (x10^9^/L), median (IQR)	4.4 (3.7-5.4)	4.3 (3.6-4.8)	4.1 (3.4-5.1)	4.3 (3.4-5.1)	0.075
Neutrophil count (x10^9^/L), median (IQR)	3.4 (2.7-4.6)	3.4 (2.6-4.2)	3.4 (2.5-4.3)	3.4 (2.7-4.3)	0.458
Lymphocyte count (x10^9^/L), median (IQR)	1.7 (1.3-2.0)	1.5 (1.3-2.0)	1.6 (1.3-2.0)	1.6 (1.3-2.0)	0.597
C-reactive protein (mg/L), median (IQR)	1.7 (0.7-4.0)	1.8 (0.8-3.4)	1.6 (0.7-3.2)	1.6 (0.6-3.3)	0.408
Hemoglobin (g/L), median (IQR)	122.0(101.0-139.0)	123.0(103.0-143.0)	125.0(106.0-139.0)	124.0 (102.0-143.0)	0.641
Cholesterol(mmol/L), median (IQR)	4.7 (4.0-5.5)	4.8 (4.1-5.7)	4.8 (4.0-5.5)	4.7 (4.0-5.3)	0.464
LDL (mmol/L), median (IQR)	2.8 (2.3-3.4)	2.8 (2.3-3.5)	2.8 (2.3-3.5)	2.9 (2.2-3.4)	0.821
HDL (mmol/L), median (IQR)	1.2 (1.0-1.4)	1.3 (1.0-1.5)	1.2 (1.0-1.5)	1.2 (1.0-1.5)	0.805
Albumin (g/L), median (IQR)	37.9 (34.8-40.5)	38.6 (36.0-42.0)	40.6 (38.2-44.0)	41.7 (38.7-45.0)	<0.001*
GNRI, median (IQR)	95.0 (90.0-99.1)	97.4 (94.4-102.5)	101.4 (98.0-105.8)	103.3 (98.9-108.2)	<0.001*
SMI (cm^2^/m^2^), median (IQR)	39.9 (34.5-42.6)	45.7 (41.9-48.6)	50.3 (44.2-52.5)	55.8 (52.8-60.0)	<0.001*
SATI (cm^2^/m^2^), median (IQR)	31.0 (21.9-44.8)	37.5 (29.9-54.0)	39.8 (28.9-53.7)	45.9 (34.0-59.7)	<0.001*
VATI (cm^2^/m^2^), median (IQR)	30.1 (18.3-44.0)	35.3 (22.1-47.5)	37.3 (24.8-54.0)	49.8 (28.2-63.3)	<0.001*
TATI (cm^2^/m^2^), median (IQR)	63.1 (45.2-86.1)	77.8 (52.1-100.6)	82.7 (61.3-101.5)	99.5 (78.7-114.6)	<0.001*
SMD (HU), median (IQR)	34.2 (29.3-41.0)	36.6 (32.5-43.2)	35.9 (31.2-41.4)	38.4 (33.0-43.0)	<0.001*

BMI, body mass index; CA19-9, carbohydrate antigen 19-9; CEA, carcinoembryonic antigen; GNRI, geriatric nutritional risk index; LDL, low-density lipoprotein; NRS-2002, nutrition risk screening-2002; POCs, Postoperative complications; HDL, high-density lipoprotein; SMI, skeletal muscle index; SATI, subcutaneous adipose tissue index; TATI, total adipose tissue index; VATI, visceral adipose tissue index; SMD, skeletal muscle density.

*P < 0.05 was considered significant.

The baseline characteristics of the patients stratified by quartiles of SMI × GNRI quartile are shown in **Table 1**. Higher SMI × GNRI values were associated with younger age, higher BMI, elevated GNRI and SMI, elevated CA19–9 levels, pathological T4 stage, and pathological N3 stage (all P < 0.05).

### 3.2 Impact of the SMI × GNRI on overall and disease‐free survival in stage II/III GC patients

In the training cohort, with a median follow-up time of 55.7 months (IQR: 44.0-67.9 months), 346 patients (50.1%) were alive, 327 (47.3%) remained disease-free, and the 1-, 3-, and 5-year mortality rates were 2.5%, 32.6%, and 51.8%, respectively. In the validation cohort, with a median follow-up of 55.0 months (IQR: 45.7-67.7 months), 147 patients (49.6%) were alive, 142 (47.9%) were disease-free, and the 1-, 3-, and 5-year mortality rates were 3.7%, 40.2%, and 60.6%, respectively.

**Figure 2:**
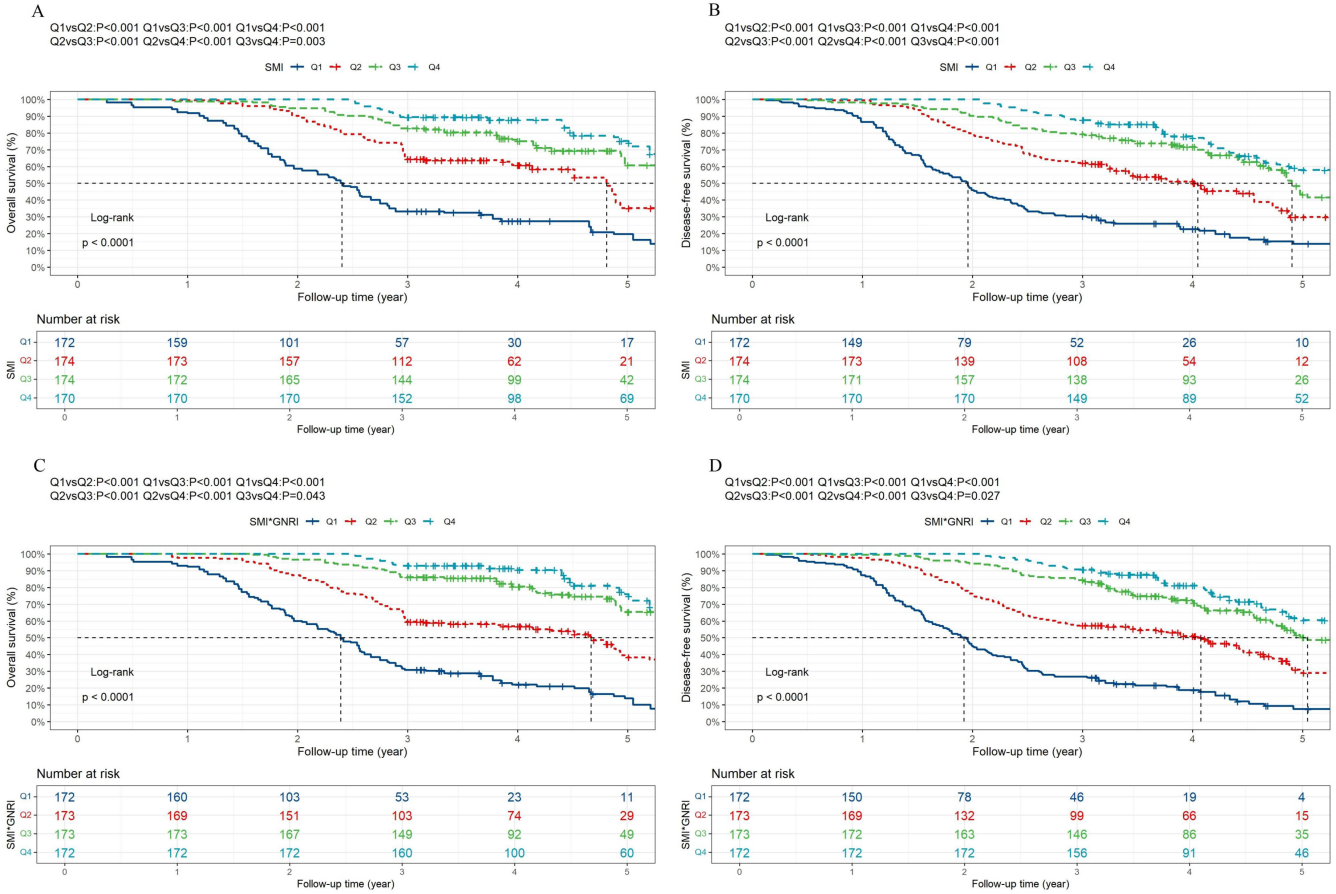
The Kaplan-Meier curves for overall survival and disease-free survival are presented for gastric cancer patients stratified according to SMI and SMI × GNRI quartiles. **(A)** Overall survival Kaplan-Meier curves of SMI. **(B)** Disease-free survival Kaplan-Meier curves of SMI. **(C)** Overall survival Kaplan-Meier curves of SMI× GNRI. **(D)** Disease-free survival Kaplan-Meier curves of SMI× GNRI. SMI, skeletal muscle index; GNRI, geriatric nutritional risk index.

**Figure 2** displays the OS and DFS curves stratified by SMI and SMI×GNRI quartiles. According to the Kaplan-Meier analysis, these SMI-based groupings demonstrated statistically significant disparities in both OS and DFS (**Figures 2A, B**; P overall < 0.0001). In addition, there were significant differences in OS between the SMI × GNRI groups (**Figure 2C**; P overall < 0.0001; Q1 vs. Q2 P < 0.001; Q1 vs. Q3 P < 0.001; Q1 vs. Q4 P < 0.001; Q2 vs. Q3 P < 0.001; Q2 vs. Q4 P < 0.001; and Q3 vs. Q4 P = 0.043). The 5-year OS rates of patients in the SMI × GNRI Q1, Q2, Q3 and Q4 groups were 13.8%, 38.3%, 65.4% and 76.1%, respectively. The median survival times for patients in the Q1, Q2, Q3, and Q4 groups were 28.7 months, 56.0 months, 67.4 months, and 70.3 months, respectively. Similarly, notable differences in DFS were also observed across the four groups (**Figure 2D**; P overall < 0.0001; Q1 vs. Q2 P < 0.001; Q1 vs. Q3 P < 0.001; Q1 vs. Q4 P < 0.001; Q2 vs. Q3 P < 0.001; Q2 vs. Q4 P < 0.001; and Q3 vs. Q4 P = 0.027).

### 3.3 Association between body composition parameters and the prognosis of patients with GC

**Figure 3:**
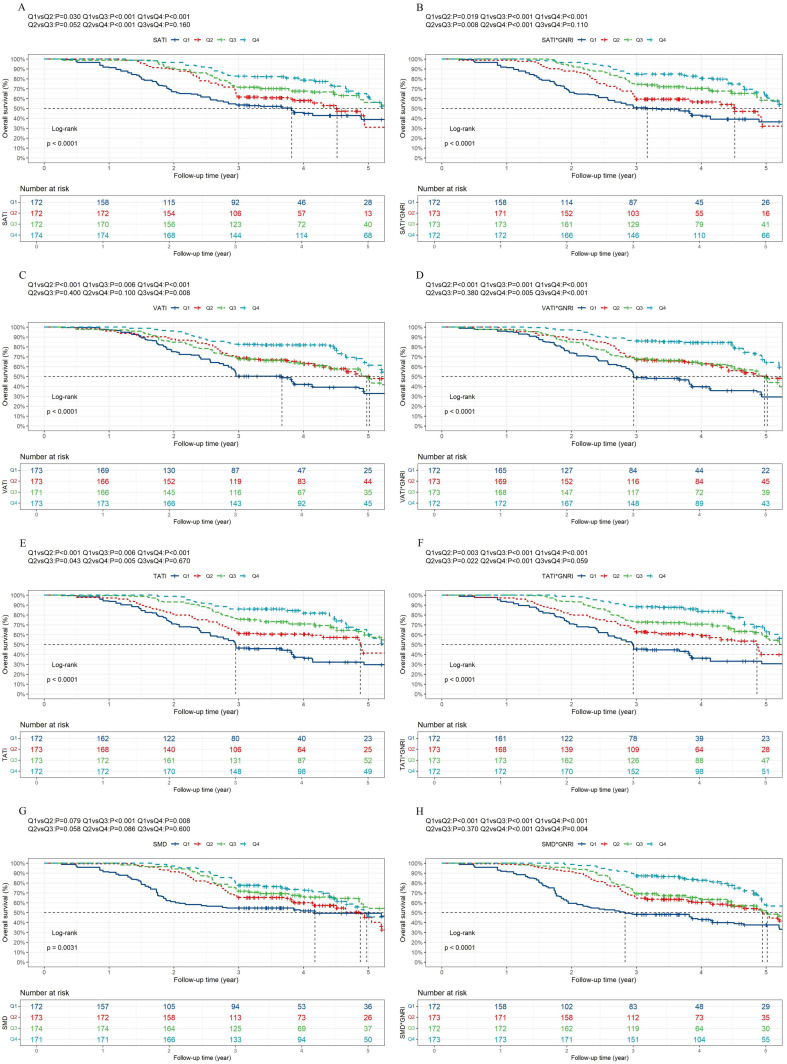
Kaplan-Meier analysis of overall survival, with patient stratification based on sex-specific quartiles of body composition parameters and corresponding GNRI-integrated indices. **(A)** Overall survival Kaplan-Meier curves of SATI. **(B)** Overall survival Kaplan-Meier curves of SATI× GNRI. **(C)** Overall survival Kaplan-Meier curves of VATI. **(D)** Overall survival Kaplan-Meier curves of VATI× GNRI. **(E)** Overall survival Kaplan-Meier curves of TATI. **(F)** Overall survival Kaplan-Meier curves of TATI× GNRI. **(G)** Overall survival Kaplan-Meier curves of SMD. **(H)** Overall survival Kaplan-Meier curves of SMD× GNRI. SATI, subcutaneous adipose tissue index; VATI, visceral adipose tissue index; TATI, total adipose tissue index; SMD, skeletal muscle density; GNRI, geriatric nutritional risk index.

**Figure 4:**
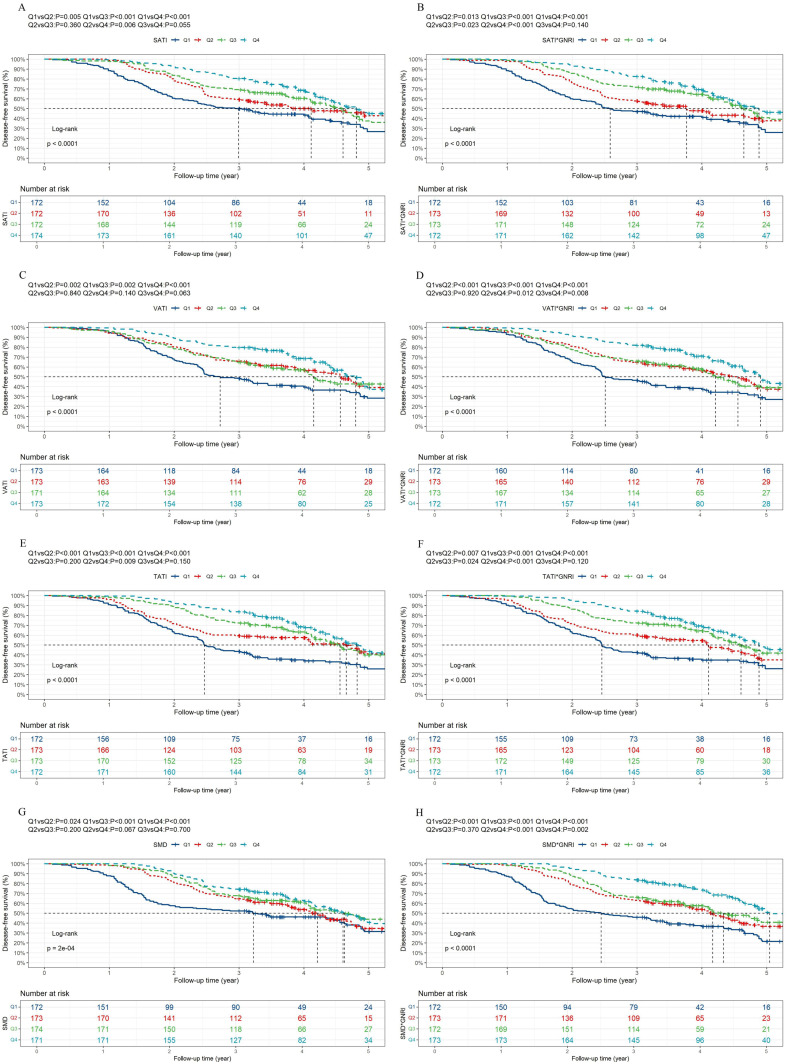
Disease-free survival Kaplan-Meier curves for patients stratified by sex-specific quartiles of body composition parameters and the combinations with GNRI. **(A)** Disease-free survival Kaplan-Meier curves of SATI. **(B)** Disease-free survival Kaplan-Meier curves of SATI× GNRI. **(C)** Disease-free survival Kaplan-Meier curves of VATI. **(D)** Disease-free survival Kaplan-Meier curves of VATI× GNRI. **(E)** Disease-free survival Kaplan-Meier curves of TATI. **(F)** Disease-free survival Kaplan-Meier curves of TATI× GNRI. **(G)** Disease-free survival Kaplan-Meier curves of SMD. **(H)** Disease-free survival Kaplan-Meier curves of SMD× GNRI. SATI, subcutaneous adipose tissue index; VATI, visceral adipose tissue index; TATI, total adipose tissue index; SMD, skeletal muscle density; GNRI, geriatric nutritional risk index.

To investigate the prognostic ability of body composition parameters and their combinations with the GNRI, we divided patients into different subgroups by sex-specific quartiles of each body composition parameter, including the SMI, SATI, VATI, TATI and SMD, and their combination with the GNRI. Univariate Cox regression analysis revealed that age, NRS-2002 score, CEA, CA19-9, pathological T stage, pathological N stage, tumor differentiation, vascular invasion, adjuvant therapy, white blood cell count, albumin and hemoglobin were significant predictors of OS (**Table 2**). In addition, age, BMI, NRS-2002 score, CEA level, pathological T stage, pathological N stage, tumor differentiation, vascular invasion, adjuvant therapy, white blood cell count, albumin and hemoglobin levels were significant predictors of DFS (**Supplementary Table 3**). Multivariate analysis showed that the associations between these body composition parameters and OS (**Tables 3**, **4**) or DFS (**Supplementary Tables 4**, **5**) remained highly significant (all P overall < 0.05). Notably, with the Q1 of the SMI × GNRI as the reference group, the hazard ratios (HRs) for OS in Q2, Q3 and Q4 were 0.451 (95% CI, 0.345-0.590), 0.201 (95% CI, 0.144-0.280) and 0.154 (95% CI, 0.106-0.224), respectively, which confirmed a robust independent association between the SMI × GNRI and OS (**Table 4**). Similarly, the SMI × GNRI was strongly and independently associated with DFS in patients with GC (overall P < 0.001; all quantile comparisons were P < 0.001; **Supplementary Table 5**). For the training cohort, the SMI × GNRI quartile cut-offs were determined as 4419, 4997, and 5511 in males and 3801, 4181, and 4825 in females. All sex-specific thresholds for the body composition parameters and their combination with the GNRI are detailed in **Supplementary Table 6**. The resulting OS and DFS curves, following stratification by these sex-specific quartiles, are presented in **Figures 3** and **4**. Given the established status of these parameters as OS risk factors, ten models were constructed. Superior prognostic performance for both OS (c-statistic: 0.749) and DFS (c-statistic: 0.731) was exhibited by the SMI×GNRI model (**Table 5**).

**Table 2:** Univariate analysis of baseline characteristics and overall survival in GC patients in the training cohort.

Variables	HR (95% CI)	P value
Age (years)	1.020 (1.002-1.038)	0.027*
Sex (Male vs. Female)	0.953 (0.763-1.190)	0.671
BMI (≥ 25 vs. < 25 kg/m^2^)	0.821 (0.657-1.027)	0.084
Hypertension (Yes vs. No)	1.168 (0.939-1.452)	0.163
Diabetes (Yes vs. No)	1.113 (0.878-1.413)	0.376
NRS-2002 (≥ 3 vs. < 3)	1.293 (1.036-1.615)	0.023*
Major POCs, n (%)	1.228 (0.907-1.662)	0.184
CEA (≥5 vs. < 5 ng/ml)	1.287 (1.019-1.626)	0.034*
CA19-9 (≥30 vs. < 30 U/L)	1.248 (1.005-1.550)	0.045*
Tumor location		
Lower and Mix	Reference	
Middle and Upper	1.143 (0.920-1.420)	0.227
Tumor size (cm)	1.029 (0.976-1.085)	0.290
Pathological T stage		<0.001*
T1	Reference	
T2	1.423 (0.711-2.848)	0.319
T3	2.167 (1.094-4.292)	0.027*
T4	2.852 (1.458-5.579)	0.002*
Pathological N stage		<0.001*
N0	Reference	
N1	0.797 (0.538-1.179)	0.256*
N2	1.546 (1.071-2.232)	0.020*
N3	2.273 (1.571-3.288)	<0.001*
Tumor differentiations		0.051
Poorly	Reference	
Moderately	0.920 (0.628-1.348)	0.668
Well	0.246 (0.079-0.768)	0.016*
Nerve invasion	1.142 (0.921-1.417)	0.226
Vascular invasion	1.349 (1.088-1.674)	0.006*
Adjuvant therapy	0.748 (0.601-0.932)	0.010*
White blood cell (x10^9^/L)	1.121 (1.041-1.207)	0.003*
Neutrophil count (x10^9^/L)	1.002 (0.964-1.041)	0.919
Lymphocyte count (x10^9^/L)	0.917 (0.767-1.095)	0.339
C-reactive protein (mg/L)	1.001 (0.989-1.012)	0.927
Hemoglobin (g/L)	0.993 (0.989-0.998)	0.003*
Cholesterol (mmol/L)	0.994 (0.903-1.093)	0.899
LDL (mmol/L)	1.008 (0.890-1.142)	0.898
HDL (mmol/L)	0.971 (0.721-1.307)	0.844
Albumin (g/L)	0.907 (0.886-0.929)	<0.001*
GNRI	0.933 (0.920-0.946)	<0.001*
SMI (cm^2^/m^2^)	0.918 (0.905-0.931)	<0.001*
SATI (cm^2^/m^2^)	0.986 (0.980-0.992)	<0.001*
VATI (cm^2^/m^2^)	0.983 (0.977-0.989)	<0.001*
TATI (cm^2^/m^2^)	0.989 (0.985-0.992)	<0.001*
SMD(HU)	0.972 (0.958-0.987)	<0.001*

BMI, body mass index; CA19-9, carbohydrate antigen 19-9; CEA, carcinoembryonic antigen; LDL, low-density lipoprotein; HDL, high-density lipoprotein; GNRI, geriatric nutritional risk index; POCs, Postoperative complications; SMI, skeletal muscle index; SATI, subcutaneous adipose tissue index; VATI, visceral adipose tissue index; TATI, total adipose tissue index; SMD, skeletal muscle density.

*P < 0.05 was considered significant.

**Table 3:** Multivariate analysis of body composition parameters and overall survival in GC patients.

Variables	Univariate analysis		Multivariate analysis									
	HR (95% CI)	P value	HR (95% CI)	P value	HR (95% CI)	P value	HR (95% CI)	P value	HR (95% CI)	P value	HR (95% CI)	P value
Age (years)	1.020 (1.002-1.038)	0.027*	1.020 (1.001-1.039)	0.042*	1.027 (1.009-1.046)	0.004*	1.034 (1.015-1.053)	<0.001*	1.028 (1.010-1.047)	0.002*	1.026 (1.008-1.045)	0.005*
NRS-2002 (≥ 3 vs. < 3)	1.293 (1.036-1.615)	0.023*	0.982 (0.714-1.352)	0.913	0.851 (0.623-1.163)	0.312	0.840 (0.618-1.140)	0.262	0.810 (0.594-1.104)	0.182	0.922 (0.679-1.253)	0.604
CEA (≥5 vs. < 5 ng/ml)	1.287 (1.019-1.626)	0.034*	1.127 (0.869-1.461)	0.367	1.337 (1.035-1.726)	0.026*	1.358 (1.051-1.754)	0.019*	1.365 (1.058-1.761)	0.016*	1.349 (1.041-1.748)	0.024*
CA19-9 (≥30 vs. < 30 U/L)	1.248 (1.005-1.550)	0.045*	1.047 (0.770-1.424)	0.769	1.217 (0.904-1.640)	0.196	1.229 (0.920-1.643)	0.163	1.173 (0.869-1.582)	0.297	1.273 (0.943-1.718)	0.115
Pathological T stage		<0.001*		<0.001*		<0.001*		<0.001*		<0.001*		<0.001*
T1	Reference		Reference		Reference		Reference		Reference		Reference	
T2	1.423 (0.711-2.848)	0.319	1.887 (0.909-3.918)	0.088	1.752 (0.849-3.616)	0.129	1.814 (0.883-3.725)	0.105	1.662 (0.807-3.421)	0.168	1.641 (0.794-3.392)	0.181
T3	2.167 (1.094-4.292)	0.027*	2.048 (0.998-4.204)	0.051	2.244 (1.101-4.573)	0.026*	2.389 (1.178-4.847)	0.016*	2.133 (1.049-4.335)	0.036*	2.251 (1.106-4.584)	0.025*
T4	2.852 (1.458-5.579)	0.002*	3.292 (1.633-6.637)	<0.001*	3.277 (1.638-6.556)	<0.001*	3.357 (1.680-6.710)	<0.001*	3.113 (1.557-6.222)	0.001*	3.112 (1.555-6.226)	0.001*
Pathological N stage		<0.001*		<0.001*		<0.001*		<0.001*		<0.001*		<0.001*
N0	Reference		Reference		Reference		Reference		Reference		Reference	
N1	0.797 (0.538-1.179)	0.256	1.616 (1.019-2.562)	0.042*	1.318 (0.857-2.028)	0.209	1.199 (0.780-1.842)	0.408	1.256 (0.817-1.930)	0.299	1.406 (0.904-2.188)	0.130
N2	1.546 (1.071-2.232)	0.020*	2.283 (1.510-3.453)	<0.001*	2.070 (1.405-3.050)	<0.001*	1.990 (1.351-2.931)	<0.001*	2.025 (1.373-2.987)	<0.001*	2.253 (1.520-3.340)	<0.001*
N3	2.273 (1.571-3.288)	<0.001*	3.103 (2.069-4.653)	<0.001*	2.768 (1.886-4.064)	<0.001*	2.704 (1.847-3.958)	<0.001*	2.756 (1.878-4.044)	<0.001*	2.953 (2.007-4.345)	<0.001*
Tumor differentiations		0.051		0.316		0.511		0.421		0.617		0.417
Poorly	Reference		Reference		Reference		Reference		Reference		Reference	
Moderately	0.920 (0.628-1.348)	0.668	0.936 (0.629-1.394)	0.745	0.892 (0.600-1.328)	0.574	1.026 (0.689-1.527)	0.901	0.982 (0.660-1.462)	0.930	0.960 (0.645-1.428)	0.840
Well	0.246 (0.079-0.768)	0.016*	0.413 (0.130-1.314)	0.134	0.545 (0.172-1.725)	0.302	0.463 (0.145-1.476)	0.193	0.561 (0.177-1.779)	0.327	0.463 (0.147-1.460)	0.189
Vascular invasion	1.349 (1.088-1.674)	0.006*	1.136 (0.909-1.419)	0.262	1.158 (0.927-1.446)	0.196	1.202 (0.964-1.499)	0.102	1.151 (0.922-1.436)	0.214	1.171 (0.939-1.460)	0.162
Adjuvant therapy	0.748 (0.601-0.932)	0.010*	0.709 (0.561-0.896)	0.004*	0.681 (0.542-0.856)	<0.001*	0.675 (0.537-0.847)	<0.001*	0.662 (0.527-0.832)	<0.001*	0.682 (0.543-0.856)	<0.001*
White blood cell (x10^9^/L)	1.121 (1.041-1.207)	0.003*	1.099 (1.016-1.188)	0.019*	1.142 (1.063-1.228)	<0.001*	1.155 (1.073-1.242)	<0.001*	1.144 (1.064-1.229)	<0.001*	1.123 (1.047-1.205)	0.001*
Hemoglobin (g/L)	0.993 (0.989-0.998)	0.003*	0.994 (0.989-0.998)	0.008*	0.993 (0.989-0.998)	0.005*	0.993 (0.988-0.998)	0.003*	0.993 (0.989-0.998)	0.004*	0.994 (0.989-0.999)	0.010*
Albumin (g/L)	0.907 (0.886-0.929)	<0.001*	0.894 (0.873-0.917)	<0.001*	0.912 (0.890-0.934)	<0.001*	0.908 (0.886-0.930)	<0.001*	0.912 (0.890-0.934)	<0.001*	0.908 (0.886-0.930)	<0.001*
SMI		<0.001*		<0.001*	–		–		–		–	
Q1	Reference		Reference		–		–		–		–	
Q2	0.418(0.319-0.547)	<0.001*	0.446(0.338-0.590)	<0.001*	–		–		–		–	
Q3	0.242(0.181-0.324)	<0.001*	0.241(0.177-0.328)	<0.001*	–		–		–		–	
Q4	0.146(0.104-0.203)	<0.001*	0.140(0.099-0.197)	<0.001*	–		–		–		–	
SATI		<0.001*	–			0.002*	–		–		–	
Q1	Reference		–		Reference		–		–		–	
Q2	0.771(0.578-1.028)	0.077	–		0.920(0.680-1.246)	0.591	–		–		–	
Q3	0.571(0.425-0.767)	<0.001*	–		0.688(0.506-0.935)	0.017*	–		–		–	
Q4	0.467(0.347-0.628)	<0.001*	–		0.582(0.428-0.793)	<0.001*	–		–		–	
VATI		<0.001*	–		–			<0.001*	–		–	
Q1	Reference		–		–		Reference		–		–	
Q2	0.593(0.445-0.791)	<0.001*	–		–		0.639(0.474-0.863)	0.003*	–		–	
Q3	0.678(0.510-0.902)	0.008*	–		–		0.717(0.532-0.965)	0.028*	–		–	
Q4	0.456(0.337-0.616)	<0.001*	–		–		0.410(0.300-0.561)	<0.001*	–		–	
TATI		<0.001*	–		–		–			<0.001*	–	
Q1	Reference		–		–		–		Reference		–	
Q2	0.611(0.460-0.812)	<0.001*	–		–		–		0.716(0.533-0.962)	0.027*	–	
Q3	0.435(0.325-0.582)	<0.001*	–		–		–		0.492(0.363-0.666)	<0.001*	–	
Q4	0.396(0.294-0.534)	<0.001*	–		–		–		0.436(0.321-0.594)	<0.001*	–	
SMD		0.004*	–		–		–		–			0.030*
Q1	Reference		–		–		–		–		Reference	
Q2	0.790(0.589-1.060)	0.116	–		–		–		–		0.887(0.654-1.205)	0.444
Q3	0.589(0.433-0.802)	<0.001*	–		–		–		–		0.709(0.512-0.981)	0.038*
Q4	0.660(0.495-0.881)	0.005*	–		–		–		–		0.665(0.491-0.900)	0.008*

CA19-9, carbohydrate antigen 19-9; CEA, carcinoembryonic antigen; NRS-2002, nutrition risk screening-2002; SMI, skeletal muscle index; SATI, subcutaneous adipose tissue index; VATI, visceral adipose tissue index; TATI, total adipose tissue index; SMD, skeletal muscle density; HR, hazard ratio; CI, confidence interval.

*P < 0.05 was considered significant.

**Table 4:** Multivariate analysis of the combination of body composition parameters and GNRI and overall survival in GC patients.

Variables	Univariate analysis		Multivariate analysis									
	HR (95% CI)	P value	HR (95% CI)	P value	HR (95% CI)	P value	HR (95% CI)	P value	HR (95% CI)	P value	HR (95% CI)	P value
Age (years)	1.020 (1.002-1.038)	0.027*	1.029 (1.011-1.048)	0.002*	1.024 (1.005-1.043)	0.014*	1.028 (1.010-1.047)	0.003*	1.027 (1.009-1.046)	0.004*	1.036 (1.017-1.055)	<0.001*
NRS-2002 (≥ 3 vs. < 3)	1.293 (1.036-1.615)	0.023*	0.833 (0.613-1.133)	0.244	0.941 (0.688-1.285)	0.700	0.834 (0.610-1.142)	0.257	0.876 (0.643-1.195)	0.403	0.845 (0.624-1.146)	0.279
CEA (≥5 vs. < 5 ng/ml)	1.287 (1.019-1.626)	0.034*	1.392 (1.080-1.794)	0.011*	1.153 (0.890-1.492)	0.281	1.364 (1.055-1.763)	0.018*	1.362 (1.050-1.768)	0.020*	1.372 (1.063-1.770)	0.015*
CA19-9 (≥30 vs. < 30 U/L)	1.248 (1.005-1.550)	0.045*	1.149 (0.855-1.544)	0.357	1.040 (0.769-1.407)	0.797	1.210 (0.897-1.633)	0.212	1.308 (0.969-1.766)	0.080	1.205 (0.903-1.608)	0.205
Pathological T stage		<0.001*		<0.001*		<0.001*		<0.001*		<0.001*		<0.001*
T1	Reference		Reference		Reference		Reference		Reference		Reference	
T2	1.423 (0.711-2.848)	0.319	1.758 (0.855-3.615)	0.125	1.939 (0.933-4.028)	0.076	1.699 (0.825-3.496)	0.150	1.527 (0.737-3.164)	0.255	1.830 (0.891-3.758)	0.100
T3	2.167 (1.094-4.292)	0.027*	2.196 (1.081-4.458)	0.030*	2.055 (1.004-4.205)	0.049*	2.171 (1.069-4.411)	0.032*	2.230 (1.096-4.538)	0.027*	2.226 (1.099-4.511)	0.026*
T4	2.852 (1.458-5.579)	0.002*	3.252 (1.629-6.492)	<0.001*	3.206 (1.595-6.446)	0.001*	3.192 (1.598-6.375)	0.001*	3.013 (1.506-6.031)	0.002*	3.344 (1.676-6.676)	<0.001*
Pathological N stage		<0.001*		<0.001*		<0.001*		<0.001*		<0.001*		<0.001*
N0	Reference		Reference		Reference		Reference		Reference		Reference	
N1	0.797 (0.538-1.179)	0.256	1.297 (0.844-1.995)	0.236	1.310 (0.837-2.052)	0.238	1.324 (0.860-2.037)	0.202	1.552 (0.996-2.419)	0.052	1.183 (0.769-1.822)	0.445
N2	1.546 (1.071-2.232)	0.020*	2.097 (1.422-3.094)	<0.001*	1.988 (1.330-2.972)	<0.001*	2.043 (1.387-3.010)	<0.001*	2.380 (1.607-3.525)	<0.001*	1.989 (1.348-2.936)	<0.001*
N3	2.273 (1.571-3.288)	<0.001*	2.849 (1.937-4.190)	<0.001*	2.707 (1.821-4.024)	<0.001*	2.753 (1.874-4.044)	<0.001*	3.133 (2.127-4.615)	<0.001*	2.744 (1.873-4.019)	<0.001*
Tumor differentiations		0.051		0.679		0.389		0.549		0.392		0.374
Poorly	Reference		Reference		Reference		Reference		Reference		Reference	
Moderately	0.920 (0.628-1.348)	0.668	0.997 (0.669-1.483)	0.986	0.887 (0.597-1.318)	0.552	0.905 (0.609-1.345)	0.622	0.952 (0.642-1.413)	0.807	1.034 (0.693-1.542)	0.871
Well	0.246 (0.079-0.768)	0.016*	0.595 (0.187-1.892)	0.379	0.475 (0.150-1.505)	0.206	0.556 (0.175-1.764)	0.319	0.452 (0.144-1.425)	0.176	0.442 (0.139-1.407)	0.167
Vascular invasion	1.349 (1.088-1.674)	0.006*	1.189 (0.951-1.487)	0.129	1.227 (0.983-1.532)	0.071	1.158 (0.926-1.448)	0.197	1.189 (0.953-1.483)	0.125	1.165 (0.932-1.454)	0.179
Adjuvant therapy	0.748 (0.601-0.932)	0.010*	0.671 (0.534-0.842)	<0.001*	0.708 (0.562-0.893)	0.004*	0.676 (0.537-0.850)	<0.001*	0.700 (0.558-0.878)	0.002*	0.666 (0.530-0.836)	<0.001*
White blood cell (x10^9^/L)	1.121 (1.041-1.207)	0.003*	1.150 (1.070-1.237)	<0.001*	1.113 (1.029-1.203)	0.007*	1.141 (1.062-1.227)	<0.001*	1.127 (1.049-1.211)	0.001*	1.150 (1.069-1.237)	<0.001*
Hemoglobin (g/L)	0.993 (0.989-0.998)	0.003*	0.994 (0.989-0.998)	0.007*	0.994 (0.989-0.998)	0.006*	0.993 (0.989-0.998)	0.004*	0.993 (0.989-0.998)	0.005*	0.994 (0.989-0.998)	0.009*
Albumin (g/L)	0.907 (0.886-0.929)	<0.001*	0.920 (0.898-0.943)	<0.001*	0.958 (0.934-0.983)	<0.001*	0.916 (0.894-0.939)	<0.001*	0.922 (0.899-0.945)	<0.001*	0.914 (0.892-0.937)	<0.001*
TATI × GNRI		<0.001*		<0.001*	–		–		–		–	
Q1	Reference		Reference		–		–		–		–	
Q2	0.663(0.503-0.874)	0.004*	0.803(0.602-1.071)	0.135	–		–		–		–	
Q3	0.461(0.345-0.616)	<0.001*	0.540(0.399-0.731)	<0.001*	–		–		–		–	
Q4	0.338(0.247-0.461)	<0.001*	0.434(0.313-0.601)	<0.001*	–		–		–		–	
SMI×GNRI		<0.001*	–			<0.001*	–		–		–	
Q1	Reference		–		Reference		–		–		–	
Q2	0.381(0.295-0.493)	<0.001*	–		0.451(0.345-0.590)	<0.001*	–		–		–	
Q3	0.161(0.117-0.220)	<0.001*	–		0.201(0.144-0.280)	<0.001*	–		–		–	
Q4	0.109(0.077-0.156)	<0.001*	–		0.154(0.106-0.224)	<0.001*	–		–		–	
SATI×GNRI		<0.001*	–		–			<0.001*	–		–	
Q1	Reference		–		–		Reference		–		–	
Q2	0.745(0.563-0.986)	0.040*	–		–		0.939(0.695-1.269)	0.683	–		–	
Q3	0.501(0.373-0.672)	<0.001*	–		–		0.618(0.455-0.838)	0.002*	–		–	
Q4	0.398(0.295-0.539)	<0.001*	–		–		0.585(0.423-0.810)	0.001*	–		–	
SMD×GNRI		<0.001*	–		–		–			<0.001*	–	
Q1	Reference		–		–		–		Reference		–	
Q2	0.587(0.444-0.778)	<0.001*	–		–		–		0.674(0.499-0.910)	0.010*	–	
Q3	0.519(0.387-0.696)	<0.001*	–		–		–		0.691(0.507-0.941)	0.019*	–	
Q4	0.356(0.264-0.481)	<0.001*	–		–		–		0.494(0.357-0.682)	<0.001*	–	
VATI×GNRI		<0.001*	–		–		–		–			<0.001*
Q1	Reference		–		–		–		–		Reference	
Q2	0.551(0.415-0.731)	<0.001*	–		–		–		–		0.613(0.457-0.821)	0.001*
Q3	0.629(0.476-0.831)	0.001*	–		–		–		–		0.735(0.548-0.985)	0.040*
Q4	0.344(0.250-0.472)	<0.001*	–		–		–		–		0.360(0.259-0.500)	<0.001*

GNRI, geriatric nutritional risk index; SMI, skeletal muscle index; SATI, subcutaneous adipose tissue index; VATI, visceral adipose tissue index; TATI, total adipose tissue index; SMD, skeletal muscle density; HR, hazard ratio; CI, confidence interval.

*P < 0.05 was considered significant.

**Table 5:** The predictive and discriminatory power of different body composition parameters and the combinations with GNRI in training cohort.

Variables	OS			DFS		
	AICc	^a^c-Statistics	*P	AICc	c-Statistics	*P
SMI	3902.3	0.722	<0.001	4231.5	0.707	<0.001
SATI	4042.1	0.620	<0.001	4356.3	0.608	<0.001
VATI	4043.2	0.602	<0.001	4362.6	0.589	<0.001
TATI	4024.5	0.644	<0.001	4342.8	0.626	<0.001
SMD	4058.2	0.596	<0.001	4368.9	0.591	<0.001
SMI×GNRI	3842.9	0.749	Reference	4174.7	0.731	Reference
SATI×GNRI	4028.3	0.640	<0.001	4345.9	0.623	<0.001
VATI×GNRI	4023.9	0.621	<0.001	4350.6	0.605	<0.001
TATI×GNRI	4015.7	0.653	<0.001	4337.7	0.633	<0.001
SMD×GNRI	4023.5	0.648	<0.001	4328.8	0.641	<0.001

OS, overall survival; DFS, disease-free survival; SMI, skeletal muscle index; SATI, subcutaneous adipose tissue index; VATI, visceral adipose tissue index; TATI, total adipose tissue index; SMD, skeletal muscle density; GNRI, geriatric nutritional risk index.

a The comparison of c-statistics was performed using variance estimation.

*P < 0.05 was considered significant.

### 3.4 Stratified analysis of the SMI × GNRI in stage II/III GC patients

To further investigate the relationship of SMI × GNRI with prognosis in stage II/III GC, stratified analyses were performed based on age, sex, BMI, NRS-2002 score, adjuvant therapy, pathological T/N stage, vascular invasion, and tumor differentiation (**Supplementary Tables 7**–**9**). Across all these subgroups, the preoperative SMI × GNRI consistently served as an independent predictor of favorable clinical outcomes, demonstrating a significant and stable positive association with survival.

### 3.5 Validation of SMI × GNRI as a prognostic tool for survival in stage II/III GC patients

In the validation cohort, which comprised 296 patients (181 men and 115 women), patient outcomes were stratified by the sex-specific quartile cut-offs from the training cohort. The subsequent analysis confirmed the SMI × GNRI model’s outperformance of all other models for predicting both OS (c-statistic: 0.734; AICc: 1441.4) and DFS (c-statistic: 0.723; AICc: 1516.6), as detailed in **Supplementary Table 10**. This result confirms the effectiveness of SMI × GNRI as an independent prognostic indicator in stage II/III GC, with corresponding survival curves detailed in **Supplementary Figures 3** and **4**.

## 4 Discussion

Through a comprehensive analysis of 986 patients, this study delineates the relationship between body composition, nutritional status, and survival in stage II/III GC patients after RS. Our findings indicate that in stage II/III GC patients, higher SMI×GNRI scores were significantly correlated with extended OS and DFS. Notably, its predictive power for OS surpassed that of all other body composition parameters - whether assessed alone or in combination with the GNRI. This association proved stable across subgroups stratified by age, sex, and other clinicopathological factors. The predictive utility of the SMI × GNRI was further validated in an independent validation cohort, underscoring its practicality as a robust prognostic indicator.

The physiological decline in muscle mass, function, and strength with aging is often compounded by malnutrition in elderly cancer patients (21). Malnutrition not only correlates with poor survival but may also foster tumor recurrence by suppressing antitumor immunity (26). The GNRI, introduced by Bouillanne et al. in 2005, serves as an objective screening tool that predicts nutrition-related morbidity and mortality, independent of inflammatory states (26, 27). Its utility in forecasting postoperative complications and OS in oncology is well-established (14, 22, 26, 28). Supporting this, Matsunaga et al. confirmed the GNRI as a predictor of OS and DFS in elderly GC patients, while a meta-analysis of 4189 GC cases linked lower preoperative GNRI to worsened prognosis (7), while a meta-analysis of 4189 GC cases linked lower preoperative GNRI to worsened prognosis (29). Notably, for stage I-III GC patients, GNRI is a key factor affecting OS irrespective of age (28). Our study aligns with these findings, demonstrating that lower GNRI scores significantly predict adverse survival in stage II/III GC patients after RS. This association may be mechanistically explained by two factors: firstly, hypoalbuminemia, a marker of chronic malnutrition and impaired immune function, is a known prognostic risk and compromises antitumor responses (30, 31). Second, weight loss, captured in the GNRI, reflects the catabolic impact of cancer and is a recognized indicator of poor prognosis (32).

Current research extensively utilizes L3-level CT cross-sectional images to assess body composition in GC (9, 33). Han et al. linked low SATI to poorer survival in cachectic patients (34). Similarly, our previous study found CT-defined low muscle mass and a high VAT/SAT ratio to be related to poor OS in locally advanced GC (9).

Significant heterogeneity is evident across studies investigating body composition in GC. For instance, one single-center study (n=101) in advanced GC patients receiving immunotherapy identified low SMI as a risk factor for early tumor regression, while high SATI correlated with more adverse events (35). A large meta-analysis (n=7615) affirmed that CT-defined low muscle mass significantly increases post-gastrectomy complication risks and reduces OS (36). Liu et al., in a study of 379 GC patients undergoing RS, observed sex-specific prognostic associations: CT-defined low muscle mass correlated with poor 5-year OS exclusively in males, while SATA was a significant factor only in females (37). These disparities may stem from physiological sex differences, such as higher testosterone levels in men promoting muscle anabolism (38). Furthermore, a separate retrospective study of 172 advanced GC patients receiving immunotherapy identified high SATA (HR = 0.42, p = 0.002) as an independent predictor of better OS, with CT-defined low muscle mass showing no prognostic value (39). The finding potentially attributable to the distinct context of advanced disease and immunotherapy, where poor nutritional status can impair immune function and weaken treatment response. Collectively, these findings underscore that the prognostic significance of body composition in GC is highly specific to the parameters evaluated (e.g., SMI, SATI) and the patient population (e.g., sex, treatment, tumor stage). Consequently, integrating body composition with other inflammatory-nutritional markers is essential for accurate risk stratification through a comprehensive assessment of patient nutritional status (39).

In the multivariate Cox regression analysis of body composition parameters and their combination with GNRI and overall survival in GC patients, we adjusted for the key clinical and pathological variables identified through univariate analysis that would affect the OS or DFS of GC patients. These variables included age, TNM stage, NRS-2002, CEA, CA19-9, tumor differentiation, tumor vascular invasion and treatment modality, among others. The purpose of this adjustment was to distinguish the independent prognostic value of combined nutritional indices of GNRI and body composition parameters from the influence of these known factors. Focusing on these primary variables of interest rather than constructing a comprehensive predictive model that includes all potential variables enabled a direct comparison of their prognostic advantages.

Integrating body composition parameters with nutritional or inflammatory indicators markedly enhances the accuracy of prognostic prediction and risk stratification. Liao et al. demonstrated that a model combining sarcopenic obesity and the lymphocyte-monocyte ratio surpassed traditional methods in predicting outcomes for hepatocellular carcinoma patients post-hepatectomy (40). Additionally, Chen et al. contributed, reporting that patients diagnosed with malnutrition by both GLIM criteria and CT-defined low muscle mass had the highest complication rates (P<0.05) and the worst survival outcomes (P < 0.0001) (41). In terms of risk stratification, Zhao et al. demonstrated that locally advanced GC patients with concurrently high PNI and SMI levels presented with significantly better OS than those with low values for both (42). Corroborating this, our study confirms that the integration of CT-defined low muscle mass and the GNRI enables precise stratification of patients with poor GC prognosis, underscoring the clinical utility of multidimensional integration.

The interplay among muscle mass, nutritional status, and chronic inflammation is pivotal in cancer cachexia (43). Single-parameter indicators often fail to represent the full systemic pathological burden. In contrast, combined indices such as the SMI × GNRI enable a simultaneous evaluation of both protein-energy status and inflammatory states, thereby creating a synergistic tool for prognostic stratification (44). Our findings support this view: multivariate analysis identified SMI, SATI, VATI, TATI, SMD, and their GNRI composite indicators as significant prognostic factors, and the SMI × GNRI indicator outperforms all others indicators in terms of predictive performance.

The rationale for employing a multiplicative, rather than an additive, model to combine SMI and GNRI is the interaction between nutritional status and body composition. We posit that malnutrition (reflected by a low GNRI) and skeletal muscle loss (reflected by a low SMI) are not merely risk factors that contribute additively to patient risk. They engage in a synergistic, vicious cycle where the presence of one condition amplifies the negative impact of the other. In this context, a multiplicative model (SMI × GNRI) better captures this biological synergy.

In constructing our multivariable models, we prioritized adjustment for the most universally accepted and robust prognostic factors in gastric cancer (age, TNM stage, and treatment modality, etc.) to clearly delineate the independent contribution of our variables of interest. Our findings regarding the superior prognostic performance of the SMI×GNRI index have direct implications for clinical practice. We propose that this readily calculable composite index be integrated into the preoperative risk assessment workflow for gastric cancer patients. Specifically, utilizing the sex-specific quartile thresholds derived from our cohort, clinicians can stratify patients into distinct risk categories (45, 46). For instance, patients falling within the lowest quartile (Q1) of SMI×GNRI should be flagged as high-risk and considered for intensive multimodal prehabilitation. Such interventions may include supervised resistance and aerobic exercise programs combined with high-protein nutritional supplementation, aimed at ameliorating their sarcopenic and nutritional status prior to major surgery (47, 48). This proactive approach could potentially enhance physiological resilience, reduce postoperative morbidity, and improve long-term survival outcomes. For patients in the intermediate quartiles (Q2-Q3), standard nutritional counseling and monitoring are advised. The SMI×GNRI index can thus serve as an objective tool within multidisciplinary team discussions to guide personalized treatment planning, including the timing of surgery and the intensity of supportive care. We acknowledge that its relative performance against other nutritional indices, such as the CONUT score or the combination of SMI and the prognostic nutritional index, remains to be determined. Future research should focus on whether targeted interventions based on SMI×GNRI index will lead to improved patient outcomes, and compare the predictive performance of these indicators to establish a hierarchy of predictive tools.

This study has several notable strengths. First, to the best of our knowledge, this is the first large-scale study to comprehensively evaluate and compare the prognostic value of combined nutritional indices consist of GNRI and body composition parameters in patients with GC. Second, our study cohort was substantial (n=986), and we employed a robust methodological approach by internally validating our findings through a 7:3 random split into training and validation cohorts, which enhanced the reliability of our results. Third, all body composition parameters were obtained from routinely available preoperative CT images within 30 days before surgery, making our proposed SMI×GNRI index a highly practical and potentially widely applicable tool that does not require additional costly examinations. Finally, our multivariable analyses were rigorously adjusted for key clinical confounders, including age, TNM stage, NRS-2002, CEA, CA19-9, tumor differentiation, tumor vascular invasion and treatment modality, strengthening the assertion of the index’s independent prognostic value.

The present study has several limitations that warrant consideration. The primary limitation is the retrospective, single-country design, which is inherently susceptible to potential selection bias and unmeasured confounding factors. For example, although we incorporate adjuvant therapy as a categorical variable (yes/no) in the multivariate cox regression analysis to control its overall impact, this binary classification cannot cover differences in specific chemotherapy regimens, cycles, or dose intensity. These factors may become unmeasured confounders. Second, while we have successfully demonstrated the predictive power and internal validity of the SMI × GNRI index using sex-specific quartiles, we also acknowledge that a universally applicable cut-off value is the ultimate goal for direct clinical applications. Furthermore, this study focused on middle-aged and elderly stage II/III GC patients at a single center, which may limit the generalizability of the findings to other settings or populations. Future multi-center studies with predefined inclusion criteria and larger, more diverse cohorts are warranted to address these limitations.

## 5 Conclusion

Our study reveals that the combination of SMI and GNRI represents a valuable prognostic indicator for the overall survival of middle-aged and elderly patients with stage II/III GC, showing better prognostic value than the rest of the body composition parameters and their combination with GNRI. SMI × GNRI is expected to be a simple and efficient tool for predicting clinical outcomes, achieving risk stratification and optimizing patient management.

## Data Availability

The raw data supporting the conclusions of this article will be made available by the authors, without undue reservation.
