# Supplementary material for: Significance of the geriatric nutritional risk index and body composition as prognostic indicators in gastric cancer patients
Source: Front Oncol. 2026 Jan 12;15:1719952. doi: 10.3389/fonc.2025.1719952 (PMC12832480; doi:10.3389/fonc.2025.1719952)
Supplement: Supplementary file 1 [file DataSheet1.docx]

Supplementary Material

# Supplementary Figures and Tables

## Supplementary Figures


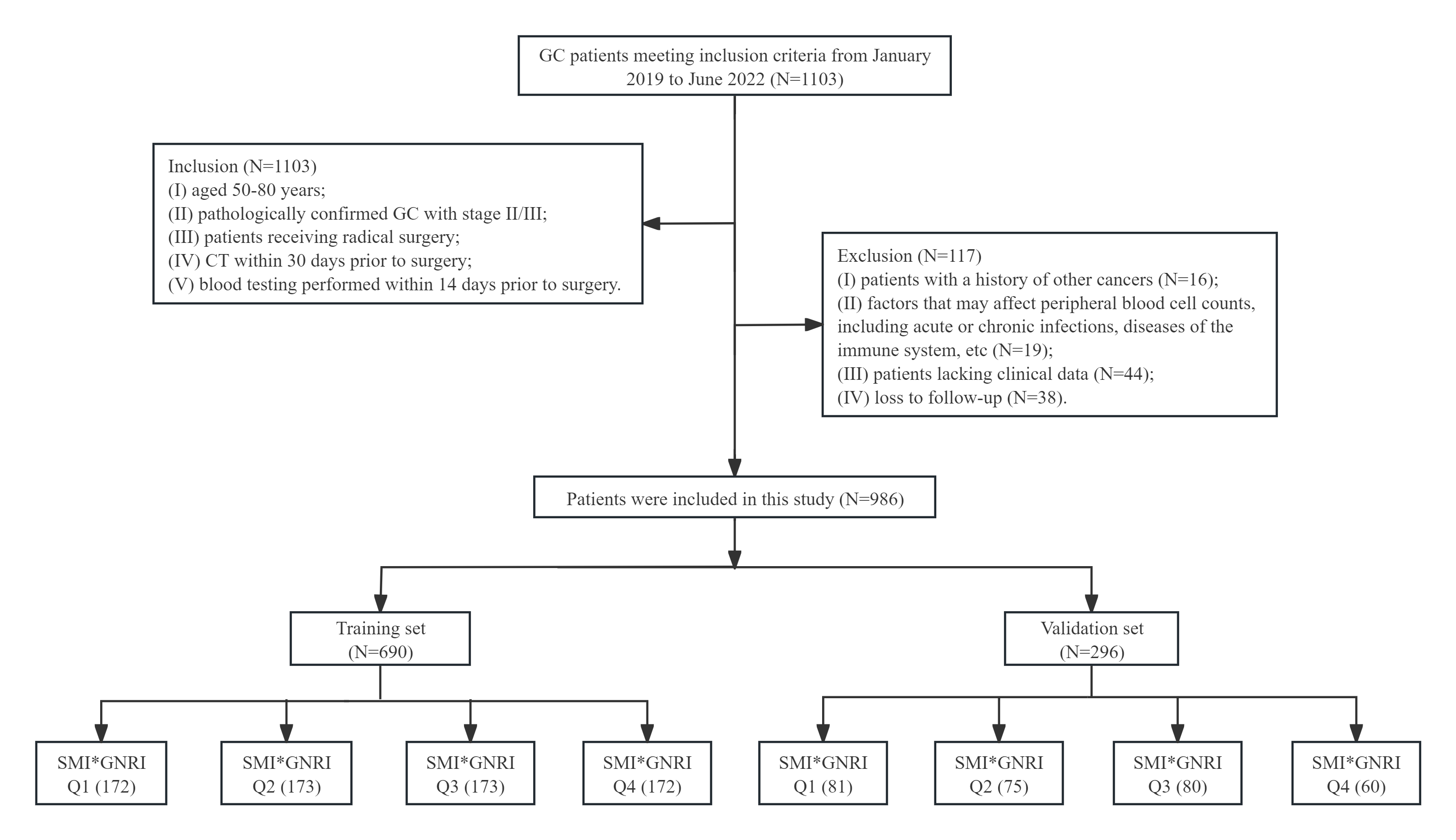


Supplementary Figure 1. Flow diagram of study design. CT, computed tomography; GC, gastric cancer; GNRI, geriatric nutritional risk index; SMI, skeletal muscle index


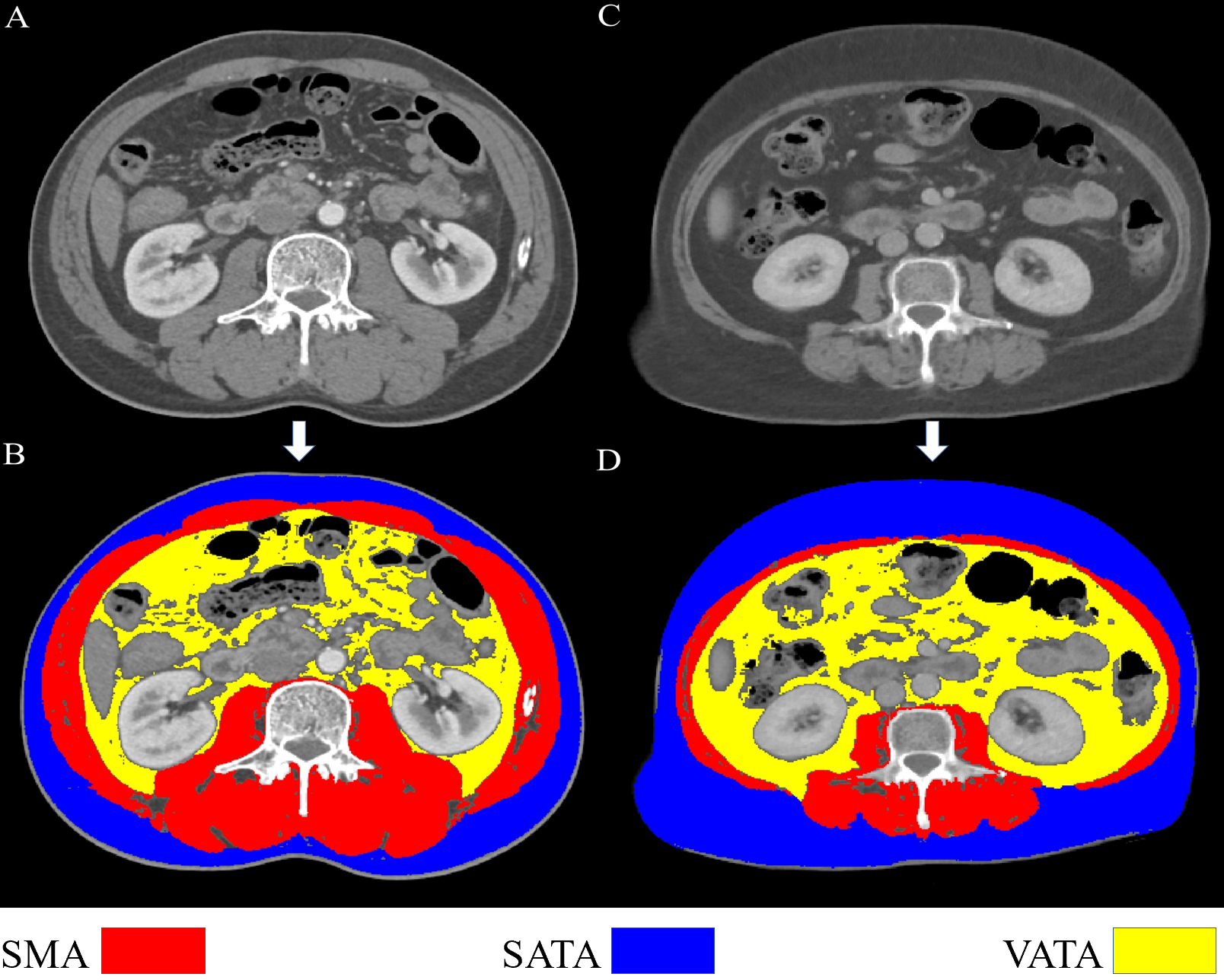


Supplementary Figure 2. Cross-sectional computed tomography images of the third lumbar vertebra used to quantify body composition parameters. The red, yellow, and blue shaded regions show the skeletal muscle, visceral adipose tissue, and subcutaneous adipose tissue areas, respectively. SMA, skeletal muscle areas; SATA, subcutaneous adipose tissue areas; VATA, visceral adipose tissue areas.

**
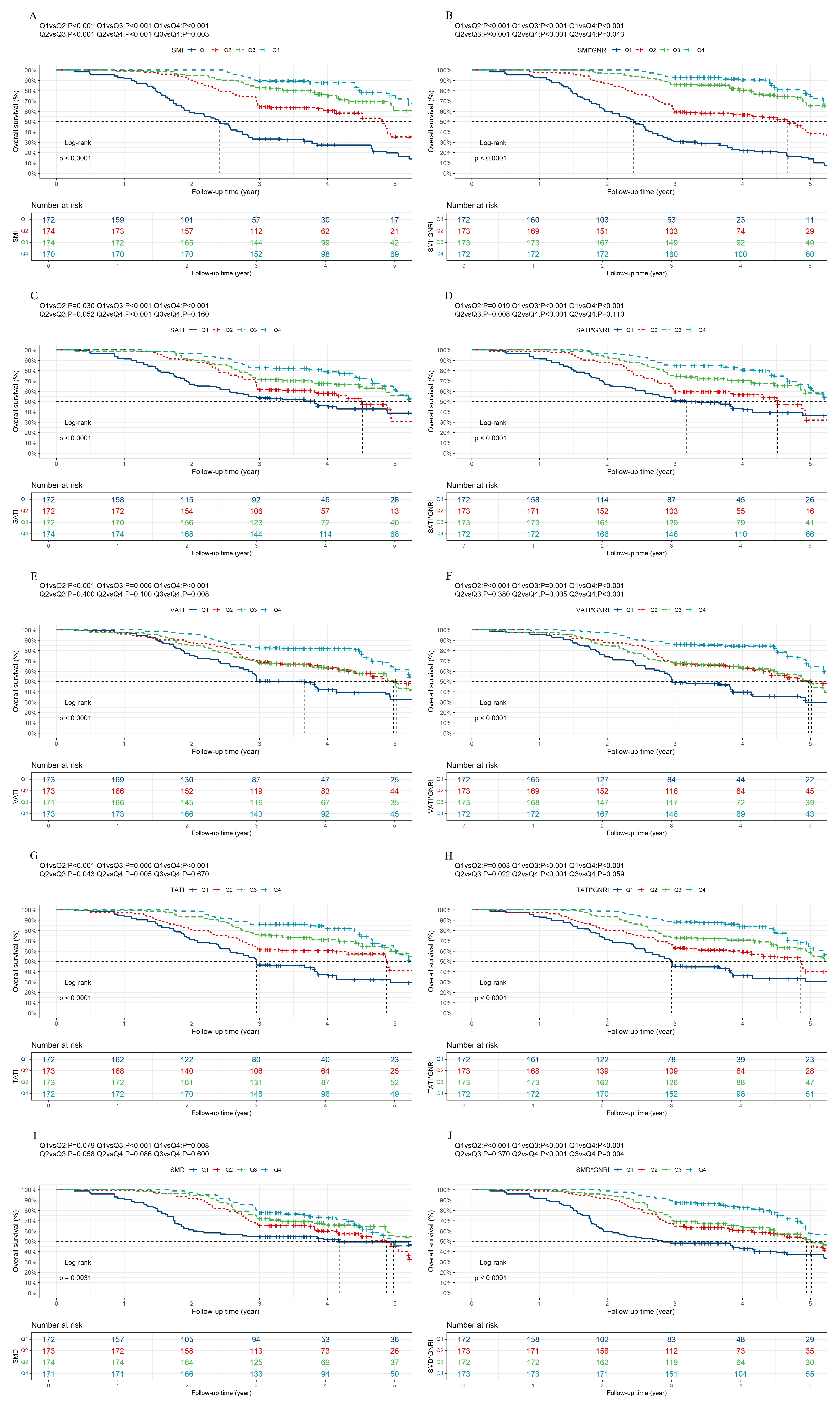
**

Supplementary Figure 3. Survival analysis of different groups of patients in the training cohort.

(A) Patients in different SMI groups (Q1-Q4). (B) Patients in different SMI×GNRI groups (Q1-Q4). (C) Patients in different SATI groups (Q1-Q4). (D) Patients in different SATI×GNRI groups (Q1-Q4). (E) Patients in different VATI groups (Q1-Q4). (F) Patients in different VATI×GNRI groups (Q1-Q4). (G) Patients in different TATI groups (Q1-Q4). (H) Patients in different TATI×GNRI groups (Q1-Q4). (I) Patients in different SMD groups (Q1-Q4). (J) Patients in different SMD×GNRI groups (Q1-Q4).

GNRI, geriatric nutritional risk index; SMI, skeletal muscle index; SATI, subcutaneous adipose tissue index; TATI, total adipose tissue index; VATI, visceral adipose tissue index; SMD, skeletal muscle density.

*P < 0.05 was considered significant.

**
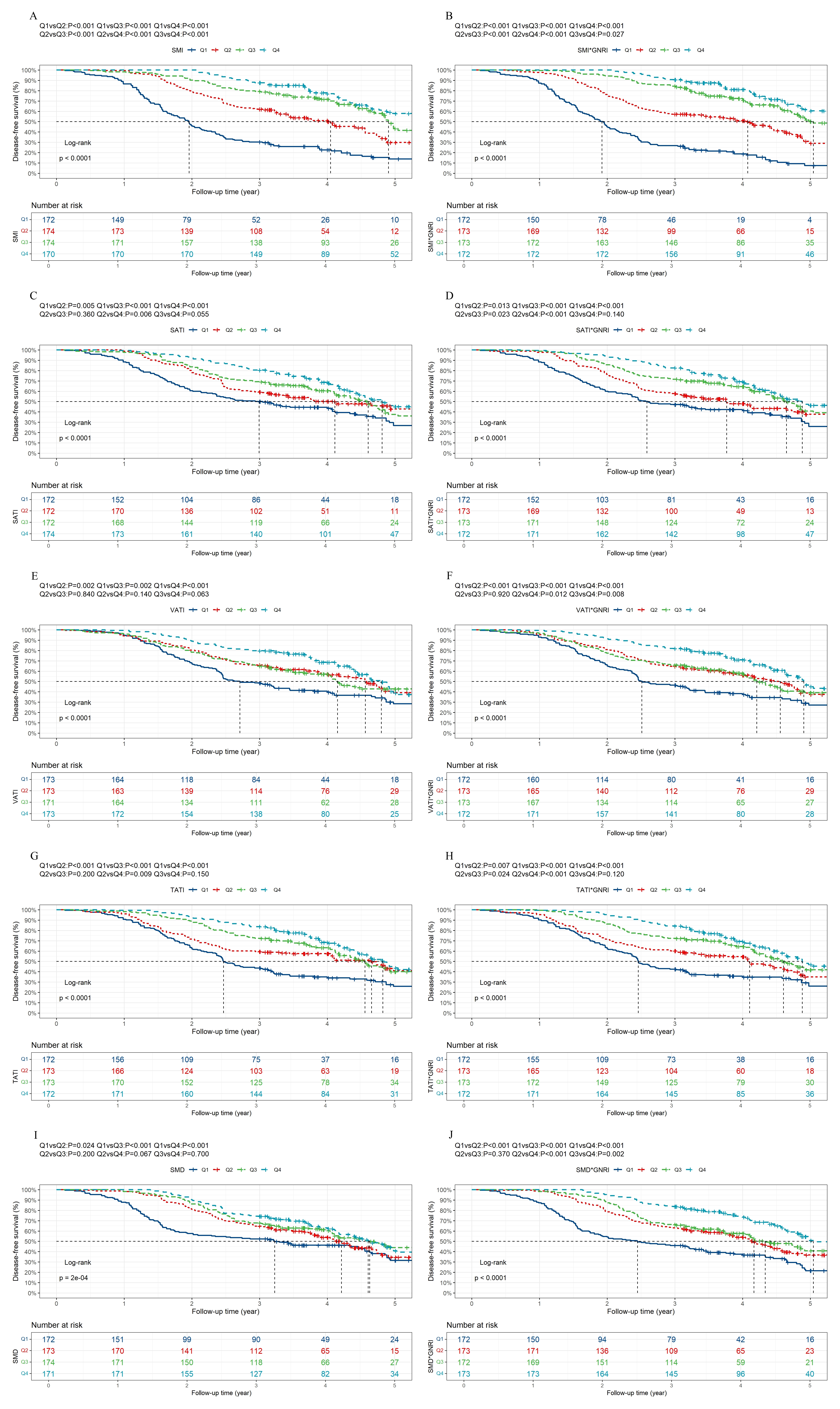
**

Supplementary Figure 4. Survival analysis of different groups of patients in the validation cohort.

(A) Patients in different SMI groups (Q1-Q4). (B) Patients in different SMI×GNRI groups (Q1-Q4). (C) Patients in different SATI groups (Q1-Q4). (D) Patients in different SATI×GNRI groups (Q1-Q4). (E) Patients in different VATI groups (Q1-Q4). (F) Patients in different VATI×GNRI groups (Q1-Q4). (G) Patients in different TATI groups (Q1-Q4). (H) Patients in different TATI×GNRI groups (Q1-Q4). (I) Patients in different SMD groups (Q1-Q4). (J) Patients in different SMD×GNRI groups (Q1-Q4).

GNRI, geriatric nutritional risk index; SMI, skeletal muscle index; SATI, subcutaneous adipose tissue index; TATI, total adipose tissue index; VATI, visceral adipose tissue index; SMD, skeletal muscle density.

*P 0.05 was considered significant.

**1.2 Supplementary Tables**

| Variables | Overall  (n=986) | Training cohort (n=690) | Validation cohort (n=296) | P value |
| --- | --- | --- | --- | --- |
| Age (years), median (IQR) | 71.0 (67.0-75.0) | 71.0 (66.0-75.0) | 71.0 (67.0-75.0) | 0.454 |
| Male gender, n (%) | 623 (63.2%) | 442 (64.1%) | 181 (61.1%) | 0.385 |
| BMI ≥ 25 (kg/m^2^), n (%) | 352 (35.7%) | 236 (37.9%) | 116 (32.0%) | 0.061 |
| Hypertension, n (%) | 341 (34.6%) | 244 (35.4%) | 97 (32.8%) | 0.433 |
| Diabetes, n (%) | 274 (27.8%) | 184 (26.7%) | 90 (30.4%) | 0.230 |
| NRS-2002≥3, n (%) | 632 (64.1%) | 441 (63.9%) | 191 (64.5%) | 0.854 |
| Major POCs, n (%) | 139 (14.1%) | 92 (13.3%) | 47 (15.9%) | 0.293 |
| CEA ≥5 (ng/ml), n (%) | 246 (24.9%) | 172 (24.9%) | 74 (25.0%) | 0.981 |
| CA19-9 ≥30 (U/L), n (%) | 573 (58.1%) | 404 (58.6%) | 169 (57.1%) | 0.671 |
| Tumor location, n (%) |  |  |  | 0.592 |
| Lower and Mix | 622 (63.1%) | 439 (63.6%) | 183 (61.8%) |  |
| Middle and Upper | 364(36.9%) | 251 (36.4%) | 113 (38.2%) |  |
| Tumor size (cm), median (IQR) | 3.5 (2.5-5.0) | 3.5 (2.5-5.0) | 3.5 (2.5-5.0) | 0.728 |
| Pathological T stage, n (%) |  |  |  | 0.564 |
| T1 | 46 (4.7%) | 33 (4.8%) | 13 (4.4%) |  |
| T2 | 254 (25.8%) | 175 (25.4%) | 79 (26.7%) |  |
| T3 | 248 (25.2%) | 182 (26.4%) | 66 (22.3%) |  |
| T4 | 438 (44.4%) | 300 (43.5%) | 138 (46.6%) |  |
| Pathological N stage, n (%) |  |  |  | 0.861 |
| N0 | 128 (13.0%) | 93 (13.5%) | 35 (11.8%) |  |
| N1 | 253 (25.7%) | 177 (25.7%) | 76 (25.7%) |  |
| N2 | 315 (31.9%) | 216 (31.3%) | 99 (33.4%) |  |
| N3 | 290 (29.4%) | 204 (29.6%) | 86 (29.1%) |  |
| Tumor differentiations, n (%) |  |  |  | 0.634 |
| Poorly | 851 (86.3%) | 600 (87.0%) | 251 (84.8%) |  |
| Moderately | 107 (10.9%) | 72 (10.4%) | 35 (11.8%) |  |
| Well | 28 (2.8%) | 18 (2.6%) | 10 (3.4%) |  |
| Nerve invasion, n (%) | 389 (39.5%) | 278 (40.3%) | 111 (37.5%) | 0.411 |
| Vascular invasion, n (%) | 540 (54.8%) | 379 (54.9%) | 161 (54.4%) | 0.877 |
| Adjuvant therapy, n (%) | 617 (62.6%) | 441 (63.9%) | 176 (59.5%) | 0.185 |
| White blood cell count (x10^9^/L), median (IQR) | 4.3 (3.5-5.2) | 4.3 (3.5-5.1) | 4.3 (3.5-5.3) | 0.819 |
| Neutrophil count (x10^9^/L), median (IQR) | 3.3 (2.6-4.3) | 3.4 (2.6-4.3) | 3.3 (2.6-4.4) | 0.547 |
| Lymphocyte count (x10^9^/L), median (IQR) | 1.6 (1.3-2.0) | 1.6 (1.3-2.0) | 1.6 (1.3-2.0) | 0.476 |
| C-reactive protein (mg/L), median (IQR) | 1.6 (0.7-3.3) | 1.7 (0.7-3.4) | 1.6 (0.6-3.1) | 0.426 |
| Hemoglobin (g/L), median (IQR) | 124.0 (104.0-140.0) | 124.0 (104.0-140.0) | 126.0 (106.0-141.0) | 0.346 |
| Cholesterol (mmol/L), median (IQR) | 4.7 (4.0-5.5) | 4.8 (4.0-5.5) | 4.7 (4.0-5.4) | 0.415 |
| LDL (mmol/L), median (IQR) | 2.8 (2.3-3.4) | 2.8 (2.3-3.4) | 2.7 (2.3-3.4) | 0.135 |
| HDL (mmol/L), median (IQR) | 1.2 (1.0-1.5) | 1.2 (1.0-1.5) | 1.2 (1.0-1.5) | 0.675 |
| Albumin (g/L), median (IQR) | 39.7 (36.9-43.0) | 39.7 (36.7-43.3) | 39.6 (37.2-42.8) | 0.698 |
| GNRI, median (IQR) | 99.2 (94.9-104.7) | 99.3 (95.0-105.0) | 99.0 (94.6-104.2) | 0.401 |
| SMI (cm^2^/m^2^), median (IQR) | 47.3 (41.7-52.5) | 47.7 (41.8-52.7) | 46.7 (41.5-52.1) | 0.074 |
| SATI (cm^2^/m^2^), median (IQR) | 38.5 (28.6-53.9) | 38.3 (28.3-53.8) | 38.8 (29.6-54.0) | 0.647 |
| VATI (cm^2^/m^2^), median (IQR) | 35.8 (24.0-51.3) | 36.2 (24.0-51.3) | 35.7 (23.9-51.8) | 0.917 |
| TATI (cm^2^/m^2^), median (IQR) | 81.8 (55.5-103.2) | 81.8 (55.0-103.3) | 82.1 (57.2-103.1) | 0.828 |
| SMD (HU), median (IQR) | 35.9 (31.3-42.5) | 35.9 (31.6-42.7) | 35.8 (30.9-42.2) | 0.342 |

Supplementary Table 1. Baseline characteristics of GC patients in our study.

BMI, body mass index; CA19-9, carbohydrate antigen 19-9; CEA, carcinoembryonic antigen; GNRI, geriatric nutritional risk index; LDL, low-density lipoprotein; NRS-2002, nutrition risk screening-2002; POCs, Postoperative complications; HDL, high-density lipoprotein; SMI, skeletal muscle index; SATI, subcutaneous adipose tissue index; TATI, total adipose tissue index; VATI, visceral adipose tissue index; SMD, skeletal muscle density.

*P < 0.05 was considered significant.

| Variables | Overall  (n=690) | Male  (n=442) | Female  (n=248) | P value |
| --- | --- | --- | --- | --- |
| Age (years) | 71.0 (66.0-75.0) | 71.0 (66.0-75.0) | 70.0 (67.0-74.0) | 0.155 |
| BMI ≥ 25 (kg/m^2^), n (%) | 263 (38.1%) | 176 (39.8%) | 87 (35.1%) | 0.219 |
| Hypertension, n (%) | 244 (35.4%) | 161 (36.4%) | 83 (33.5%) | 0.436 |
| Diabetes, n (%) | 184 (26.7%) | 108 (24.4%) | 76 (30.6%) | 0.077 |
| NRS-2002 ≥ 3, n (%) | 441 (63.9%) | 292 (66.1%) | 149 (60.1%) | 0.116 |
| Major POCs, n (%) | 92 (13.3%) | 55 (12.4%) | 37 (14.9%) | 0.359 |
| CEA ≥5 (ng/ml), n (%) | 172 (24.9%) | 112 (25.3%) | 60 (24.2%) | 0.738 |
| CA19-9 ≥30 (U/L), n (%) | 404 (58.6%) | 264 (59.7%) | 140 (56.5%) | 0.402 |
| Tumor location, n (%) |  |  |  | 0.646 |
| Lower and Mix | 439 (63.6%) | 284 (64.3%) | 155 (62.5%) |  |
| Middle and Upper | 251 (36.4%) | 158 (35.7%) | 93 (37.5%) |  |
| Tumor size, cm | 3.5 (2.5-5.0) | 3.5 (2.5-5.0) | 3.7 (3.0-5.0) | 0.122 |
| Pathological T stage, n (%) |  |  |  | 0.720 |
| T1 | 33 (4.8%) | 23 (5.2%) | 10 (4.0%) |  |
| T2 | 175 (25.4%) | 109 (24.7%) | 66 (26.6%) |  |
| T3 | 182 (26.4%) | 113 (25.6%) | 69 (27.8%) |  |
| T4 | 300 (43.5%) | 197 (44.6%) | 103 (41.5%) |  |
| Pathological N stage, n (%) |  |  |  | 0.889 |
| N0 | 93 (13.5%) | 59 (13.3%) | 34 (13.7%) |  |
| N1 | 177 (25.7%) | 117 (26.5%) | 60 (24.2%) |  |
| N2 | 216 (31.3%) | 139 (31.4%) | 77 (31.0%) |  |
| N3 | 204 (29.6%) | 127 (28.7%) | 77 (31.0%) |  |
| Tumor differentiations, n (%) |  |  |  | 0.174 |
| Poorly | 600 (87.0%) | 384 (86.9%) | 216 (87.1%) |  |
| Moderately | 72 (10.4%) | 43 (9.7%) | 29 (11.7%) |  |
| Well | 18 (2.6%) | 15 (3.4%) | 3 (1.2%) |  |
| Nerve invasion, n (%) | 278 (40.3%) | 185 (41.9%) | 93 (37.5%) | 0.263 |
| Vascular invasion, n (%) | 379 (54.9%) | 242 (54.8%) | 137 (55.2%) | 0.901 |
| Adjuvant therapy, n (%) | 441 (63.9%) | 286 (64.7%) | 155 (62.5%) | 0.563 |
| White blood cell count  (x10^9^/L), median(IQR) | 4.3 (3.5-5.1) | 4.4 (3.5-5.2) | 4.2 (3.4-5.0) | 0.135 |
| Neutrophil count (x10^9^/L), median(IQR) | 3.4 (2.6-4.3) | 3.5 (2.6-4.3) | 3.2 (2.6-4.3) | 0.238 |
| Lymphocyte count (x10^9^/L), median (IQR) | 1.6 (1.3-2.0) | 1.6 (1.3-2.0) | 1.6 (1.3-2.0) | 0.890 |
| 1. reactive protein (mg/L), median (IQR) | 1.7 (0.7-3.4) | 1.6 (0.6-3.6) | 1.8 (1.0-3.3) | 0.310 |
| Hemoglobin (g/L), median (IQR) | 124.0(104.0-140.0) | 124.0(104.0-141.0) | 123.0(102.0-138.0) | 0.444 |
| Cholesterol (mmol/L), median (IQR) | 4.8 (4.0-5.5) | 4.8 (4.0-5.6) | 4.8 (4.0-5.6) | 0.514 |
| LDL (mmol/L), median (IQR) | 2.8 (2.3-3.4) | 2.8 (2.3-3.5) | 2.8 (2.3-3.5) | 0.578 |
| HDL (mmol/L), median (IQR) | 1.2 (1.0-1.5) | 1.2 (1.0-1.5) | 1.2 (1.0-1.5) | 0.695 |
| Albumin (g/L), median (IQR) | 39.7(36.7-43.3) | 40.0(37.3-43.3) | 39.2(36.1-43.2) | 0.064 |
| GNRI, median (IQR) | 99.3(95.0-105.0) | 99.9(95.5-105.0) | 98.1(94.4-105.0) | 0.069 |
| SMI (cm^2^/m^2^), median (IQR) | 47.7 (41.8-52.7) | 50.2 (44.3-53.7) | 41.9 (39.8-49.4) | <0.001^*^ |
| SATI (cm^2^/m^2^), median (IQR) | 38.3 (28.3-53.8) | 36.0 (25.2-50.0) | 47.4 (34.4-63.6) | <0.001^*^ |
| VATI (cm^2^/m^2^), median (IQR) | 36.2 (24.0-51.3) | 40.4 (24.6-54.7) | 32.8 (22.1-45.2) | <0.001^*^ |
| TATI (cm^2^/m^2^), median (IQR) | 81.8(55.0-103.3) | 79.8(49.5-102.9) | 85.5(63.2-103.9) | 0.005^*^ |
| SMD (HU), median (IQR) | 35.9 (31.6-42.7) | 39.1 (33.9-44.2) | 32.1 (28.7-36.3) | <0.001^*^ |

Supplementary Table 2. Baseline characteristics of GC patients in the training cohort.

BMI, body mass index; CA19-9, carbohydrate antigen 19-9; CEA, carcinoembryonic antigen; GNRI, geriatric nutritional risk index; LDL, low-density lipoprotein; NRS-2002, nutrition risk screening-2002; POCs, Postoperative complications; HDL, high-density lipoprotein; SMI, skeletal muscle index; SATI, subcutaneous adipose tissue index; TATI, total adipose tissue index; VATI, visceral adipose tissue index; SMD, skeletal muscle density.

*P < 0.05 was considered significant.

| Variables | HR (95% CI) | P value |
| --- | --- | --- |
| Age (years) | 1.019 (1.001-1.036) | 0.034* |
| Sex (Male vs. Female) | 0.931 (0.752-1.153) | 0.514 |
| BMI (≥ 25 vs. < 25 kg/m^2^) | 0.804 (0.648-0.997) | 0.047* |
| Hypertension (Yes vs. No) | 1.059 (0.855-1.312) | 0.598 |
| Diabetes (Yes vs. No) | 1.071 (0.849-1.352) | 0.562 |
| NRS-2002 (≥ 3 vs. < 3) | 1.271 (1.024-1.578) | 0.029* |
| Major POCs, n (%) | 1.007 (0.739-1.372) | 0.965 |
| CEA (≥5 vs. < 5 ng/ml) | 1.271 (1.010-1.599) | 0.041* |
| CA19-9 (≥30 vs. < 30 U/L) | 1.206 (0.978-1.488) | 0.080 |
| Tumor location |  |  |
| Lower and Mix | Reference |  |
| Middle and Upper | 1.046 (0.845-1.295) | 0.679 |
| Tumor size (cm) | 1.007 (0.956-1.060) | 0.794 |
| Pathological T stage |  | <0.001* |
| T1 | Reference |  |
| T2 | 1.303 (0.672-2.525) | 0.434 |
| T3 | 2.281 (1.192-4.364) | 0.013* |
| T4 | 2.810 (1.486-5.315) | 0.001* |
| Pathological N stage |  | <0.001* |
| N0 | Reference |  |
| N1 | 0.784 (0.532-1.156) | 0.220 |
| N2 | 1.606 (1.120-2.303) | 0.010* |
| N3 | 2.385 (1.660-3.425) | <0.001* |
| Tumor differentiations |  | 0.044* |
| Poorly | Reference |  |
| Moderately | 0.878 (0.611-1.263) | 0.484 |
| Well | 0.246 (0.079-0.767) | 0.016* |
| Nerve invasion | 1.149 (0.932-1.416) | 0.195 |
| Vascular invasion | 1.256 (1.019-1.548) | 0.033* |
| Adjuvant therapy | 0.759 (0.613-0.940) | 0.011* |
| White blood cell count(x10^9^/L) | 1.115 (1.037-1.198) | 0.003* |
| Neutrophil count (x10^9^/L) | 1.006 (0.974-1.038) | 0.719 |
| Lymphocyte count (x10^9^/L) | 0.961 (0.808-1.142) | 0.649 |
| C-reactive protein (mg/L) | 1.001 (0.990-1.012) | 0.821 |
| Hemoglobin (g/L) | 0.994 (0.990-0.998) | 0.004* |
| Cholesterol (mmol/L) | 0.971 (0.885-1.066) | 0.540 |
| LDL (mmol/L) | 0.964 (0.853-1.088) | 0.551 |
| HDL (mmol/L) | 0.958 (0.717-1.281) | 0.773 |
| Albumin (g/L) | 0.916 (0.895-0.937) | <0.001* |
| GNRI | 0.937 (0.924-0.950) | <0.001* |
| SMI (cm^2^/m^2^) | 0.923 (0.910-0.936) | <0.001* |
| SATI (cm^2^/m^2^) | 0.987 (0.982-0.993) | <0.001* |
| VATI (cm^2^/m^2^) | 0.985 (0.979-0.991) | <0.001* |
| TATI (cm^2^/m^2^) | 0.990 (0.986-0.993) | <0.001* |
| SMD（HU） | 0.967 (0.953-0.982) | <0.001* |

Supplementary Table 3 . Univariate analysis of baseline characteristics and disease-free survival in GC patients in the training cohort.

BMI, body mass index; CA19-9, carbohydrate antigen 19-9; CEA, carcinoembryonic antigen; LDL, low-density lipoprotein; NRS-2002, nutrition risk screening-2002; POCs, Postoperative complications; HDL, high-density lipoprotein; GNRI, geriatric nutritional risk index; SMI, skeletal muscle index; SATI, subcutaneous adipose tissue index; VATI, visceral adipose tissue index; TATI, total adipose tissue index; SMD, skeletal muscle density.

*P < 0.05 was considered significant.

| Variables | Univariate analysis | | Multivariate analysis | | | | | | | | | |
| --- | --- | --- | --- | --- | --- | --- | --- | --- | --- | --- | --- | --- |
|  | HR (95% CI) | P value | HR (95% CI) | P value | HR (95% CI) | P value | HR (95% CI) | P value | HR (95% CI) | P value | HR (95% CI) | P value |
| Age (years) | 1.019 (1.001-1.036) | 0.034* | 1.022 (1.003-1.040) | 0.021* | 1.025 (1.007-1.043) | 0.006* | 1.032 (1.014-1.050) | <0.001* | 1.028 (1.010-1.046) | 0.002* | 1.024 (1.006-1.042) | 0.009* |
| BMI (≥ 25 vs. < 25 kg/m^2^) | 0.804 (0.648-0.997) | 0.047* | 1.345 (1.058-1.710) | 0.015* | 0.989 (0.776-1.259) | 0.926 | 1.091 (0.854-1.393) | 0.487 | 1.209 (0.934-1.565) | 0.149 | 0.779 (0.625-0.973) | 0.027* |
| NRS-2002 (≥ 3 vs. < 3) | 1.271 (1.024-1.578) | 0.029* | 0.971 (0.760-1.241) | 0.814 | 0.936 (0.735-1.191) | 0.589 | 0.933 (0.733-1.187) | 0.573 | 0.877 (0.689-1.115) | 0.283 | 1.037(0.817-1.317) | 0.763 |
| CEA (≥5 vs. < 5 ng/ml) | 1.271 (1.010-1.599) | 0.041* | 1.154 (0.900-1.481) | 0.258 | 1.377 (1.076-1.762) | 0.011* | 1.429 (1.115-1.830) | 0.005* | 1.423 (1.114-1.819) | 0.005* | 1.402 (1.093-1.797) | 0.008* |
| Pathological T stage |  | <0.001* |  | <0.001* |  | <0.001* |  | <0.001* |  | <0.001* |  | <0.001* |
| T1 | Reference |  | Reference |  | Reference |  | Reference |  | Reference |  | Reference |  |
| T2 | 1.303 (0.672-2.525) | 0.434 | 1.834 (0.915-3.675) | 0.087 | 1.714 (0.861-3.414) | 0.125 | 1.723 (0.868-3.423) | 0.120 | 1.629 (0.819-3.238) | 0.164 | 1.594 (0.797-3.187) | 0.187 |
| T3 | 2.281 (1.192-4.364) | 0.013* | 2.545 (1.289-5.026) | 0.007* | 2.565 (1.307-5.034) | 0.006* | 2.646 (1.353-5.172) | 0.004* | 2.504 (1.279-4.902) | 0.007* | 2.509 (1.278-4.926) | 0.008* |
| T4 | 2.810 (1.486-5.315) | 0.001* | 3.407 (1.749-6.636) | <0.001* | 3.402 (1.760-6.573) | <0.001* | 3.336 (1.727-6.443) | <0.001* | 3.226 (1.670-6.229) | <0.001* | 3.152 (1.629-6.096) | <0.001* |
| Pathological N stage |  | <0.001* |  | <0.001* |  | <0.001* |  | <0.001* |  | <0.001* |  | <0.001* |
| N0 | Reference |  | Reference |  | Reference |  | Reference |  | Reference |  | Reference |  |
| N1 | 0.784 (0.532-1.156) | 0.220 | 1.397 (0.899-2.173) | 0.137 | 1.225 (0.803-1.869) | 0.345 | 1.132 (0.742-1.726) | 0.566 | 1.204 (0.789-1.837) | 0.389 | 1.342 (0.873-2.061) | 0.180 |
| N2 | 1.606 (1.120-2.303) | 0.010* | 2.255 (1.526-3.332) | <0.001* | 2.265 (1.552-3.306) | <0.001* | 2.151 (1.475-3.137) | <0.001* | 2.218 (1.518-3.239) | <0.001* | 2.407 (1.645-3.522) | <0.001* |
| N3 | 2.385 (1.660-3.425) | <0.001* | 2.999 (2.045-4.399) | <0.001* | 2.770 (1.908-4.020) | <0.001* | 2.778 (1.917-4.025) | <0.001* | 2.866 (1.973-4.164) | <0.001* | 2.904 (2.001-4.214) | <0.001* |
| Tumor differentiations |  | 0.044* |  | 0.151 |  | 0.370 |  | 0.414 |  | 0.458 |  | 0.372 |
| Poorly | Reference |  | Reference |  | Reference |  | Reference |  | Reference |  | Reference |  |
| Moderately | 0.878 (0.611-1.263) | 0.484 | 0.833 (0.567-1.222) | 0.350 | 0.849 (0.581-1.241) | 0.398 | 0.952 (0.653-1.389) | 0.800 | 0.903 (0.620-1.317) | 0.597 | 0.948 (0.649-1.384) | 0.781 |
| Well | 0.246 (0.079-0.767) | 0.016* | 0.355 (0.112-1.127) | 0.079 | 0.506 (0.160-1.597) | 0.245 | 0.462 (0.146-1.463) | 0.189 | 0.508 (0.161-1.602) | 0.248 | 0.443 (0.141-1.396) | 0.165 |
| Vascular invasion | 1.256 (1.019-1.548) | 0.033* | 1.091 (0.880-1.351) | 0.428 | 1.090 (0.880-1.351) | 0.431 | 1.146 (0.925-1.419) | 0.212 | 1.103 (0.891-1.366) | 0.366 | 1.104 (0.891-1.369) | 0.365 |
| Adjuvant therapy | 0.759 (0.613-0.940) | 0.011* | 0.748 (0.598-0.937) | 0.011* | 0.719 (0.576-0.897) | 0.003* | 0.702 (0.563-0.875) | 0.002* | 0.694 (0.556-0.866) | 0.001* | 0.707 (0.567-0.881) | 0.002* |
| White blood cell (x10^9^/L) | 1.115 (1.037-1.198) | 0.003* | 1.087 (1.007-1.172) | 0.032* | 1.142 (1.063-1.226) | <0.001* | 1.148 (1.069-1.233) | <0.001* | 1.138 (1.060-1.221) | <0.001* | 1.124 (1.049-1.205) | <0.001* |
| Hemoglobin (g/L) | 0.994 (0.990-0.998) | 0.004* | 0.995 (0.990-0.999) | 0.017* | 0.994 (0.990-0.998) | 0.008* | 0.993 (0.989-0.998) | 0.003* | 0.994 (0.989-0.998) | 0.004* | 0.994 (0.989-0.998) | 0.004* |
| Albumin (g/L) | 0.916 (0.895-0.937) | <0.001* | 0.902 (0.881-0.924) | <0.001* | 0.919 (0.898-0.941) | < 0.001* | 0.915 (0.894-0.937) | <0.001* | 0.920 (0.899-0.942) | <0.001* | 0.914 (0.893-0.936) | <0.001* |
| SMI |  | <0.001* |  | <0.001* | - | | - | | - | | - | |
| Q1 | Reference |  | Reference |  | - | | - | | - | | - | |
| Q2 | 0.425 (0.327-0.552) | <0.001* | 0.456 (0.348-0.597) | <0.001* | - | | - | | - | | - | |
| Q3 | 0.245 (0.184-0.326) | <0.001* | 0.241 (0.178-0.327) | <0.001* | - | | - | | - | | - | |
| Q4 | 0.172 (0.125-0.235) | <0.001* | 0.144 (0.102-0.204) | <0.001* | - | | - | | - | | - | |
| SATI |  | <0.001* | - | |  | 0.002* | - | | - | | - | |
| Q1 | Reference |  | - | | Reference |  | - | | - | | - | |
| Q2 | 0.671 (0.506-0.890) | 0.006* | - | | 0.765 (0.566-1.033) | 0.080 | - | | - | | - | |
| Q3 | 0.581 (0.438-0.772) | <0.001* | - | | 0.686 (0.501-0.940) | 0.019* | - | | - | | - | |
| Q4 | 0.451 (0.339-0.601) | <0.001* | - | | 0.531 (0.385-0.731) | <0.001* | - | | - | | - | |
| VATI |  | <0.001* | - | | - | |  | <0.001* | - | | - | |
| Q1 | Reference |  | - | | - | | Reference |  | - | | - | |
| Q2 | 0.623 (0.472-0.824) | <0.001* | - | | - | | 0.660 (0.492-0.885) | 0.006* | - | | - | |
| Q3 | 0.643 (0.485-0.852) | 0.002* | - | | - | | 0.654 (0.484-0.883) | 0.006* | - | | - | |
| Q4 | 0.490 (0.366-0.655) | <0.001* | - | | - | | 0.428 (0.306-0.598) | <0.001* | - | | - | |
| TATI |  | <0.001* | - | | - | | - | |  | <0.001* | - | |
| Q1 | Reference |  | - | | - | | - | | Reference |  | - | |
| Q2 | 0.596 (0.451-0.789) | <0.001* | - | | - | | - | | 0.643 (0.479-0.863) | 0.003* | - | |
| Q3 | 0.488 (0.369-0.645) | <0.001* | - | | - | | - | | 0.498 (0.366-0.677) | <0.001* | - | |
| Q4 | 0.394 (0.294-0.527) | <0.001* | - | | - | | - | | 0.373 (0.263-0.530) | <0.001* | - | |
| SMD |  | <0.001* | - | | - | | - | | - | |  | 0.001* |
| Q1 | Reference |  | - | | - | | - | | - | | Reference |  |
| Q2 | 0.720 (0.543-0.955) | 0.023* | - | | - | | - | | - | | 0.813 (0.607-1.090) | 0.166 |
| Q3 | 0.590 (0.442-0.789) | <0.001* | - | | - | | - | | - | | 0.698 (0.515-0.946) | 0.020* |
| Q4 | 0.566 (0.425-0.754) | 0.005* | - | | - | | - | | - | | 0.553 (0.410-0.746) | <0.001* |

Supplementary Table 4. Multivariate analysis of body composition parameters and disease-free survival in GC patients.

BMI, body mass index; CEA, carcinoembryonic antigen; NRS-2002, nutrition risk screening-2002; SMI, skeletal muscle index; SATI, subcutaneous adipose tissue index; VATI, visceral adipose tissue index; TATI, total adipose tissue index; SMD, skeletal muscle density; HR, hazard ratio; CI, confidence interval.

*P < 0.05 was considered significant.

| Variables | Univariate analysis | | Multivariate analysis | | | | | | | | | |
| --- | --- | --- | --- | --- | --- | --- | --- | --- | --- | --- | --- | --- |
|  | HR (95% CI) | P value | HR (95% CI) | P value | HR (95% CI) | P value | HR (95% CI) | P value | HR (95% CI) | P value | HR (95% CI) | P value |
| Age (years) | 1.019 (1.001-1.036) | 0.034* | 1.025 (1.007-1.044) | 0.006* | 1.026 (1.008-1.045) | 0.004* | 1.033 (1.015-1.052) | <0.001* | 1.028 (1.010-1.047) | 0.002* | 1.024 (1.006-1.042) | 0.009* |
| BMI (≥ 25 vs. < 25 kg/m^2^) | 0.804 (0.648-0.997) | 0.047* | 1.379 (1.083-1.757) | 0.009* | 1.022 (0.799-1.308) | 0.862 | 1.124 (0.878-1.439) | 0.353 | 1.169 (0.904-1.511) | 0.235 | 0.782 (0.627-0.975) | 0.029* |
| NRS-2002 (≥ 3 vs. < 3) | 1.271 (1.024-1.578) | 0.029* | 0.895 (0.703-1.139) | 0.368 | 0.916 (0.718-1.169) | 0.481 | 0.929 (0.730-1.183) | 0.550 | 0.887 (0.697-1.128) | 0.329 | 0.995 (0.783-1.266) | 0.970 |
| CEA (≥5 vs. < 5 ng/ml) | 1.271 (1.010-1.599) | 0.041* | 1.175 (0.918-1.503) | 0.200 | 1.405 (1.097-1.799) | 0.007* | 1.446 (1.130-1.849) | 0.003* | 1.447 (1.133-1.849) | 0.003* | 1.438 (1.119-1.847) | 0.005* |
| Pathological T stage |  | <0.001* |  | <0.001* |  | <0.001* |  | <0.001* |  | <0.001* |  | <0.001* |
| T1 | Reference |  | Reference |  | Reference |  | Reference |  | Reference |  | Reference |  |
| T2 | 1.303 (0.672-2.525) | 0.434 | 1.878 (0.936-3.768) | 0.076 | 1.649 (0.830-3.276) | 0.153 | 1.765 (0.889-3.508) | 0.105 | 1.662 (0.837-3.301) | 0.147 | 1.470 (0.735-2.943) | 0.276 |
| T3 | 2.281 (1.192-4.364) | 0.013* | 2.570 (1.304-5.063) | 0.006* | 2.471 (1.263-4.834) | 0.008* | 2.557 (1.309-4.995) | 0.006* | 2.481 (1.268-4.854) | 0.008* | 2.435 (1.242-4.774) | 0.010* |
| T4 | 2.810 (1.486-5.315) | 0.001* | 3.413 (1.753-6.646) | <0.001* | 3.300 (1.710-6.367) | <0.001* | 3.376 (1.750-6.514) | <0.001* | 3.268 (1.695-6.303) | <0.001* | 2.990 (1.545-5.785) | 0.001* |
| Pathological N stage |  | <0.001* |  | <0.001* |  | <0.001* |  | <0.001* |  | <0.001* |  | <0.001* |
| N0 | Reference |  | Reference |  | Reference |  | Reference |  | Reference |  | Reference |  |
| N1 | 0.784 (0.532-1.156) | 0.220 | 1.236 (0.801-1.909) | 0.338 | 1.241 (0.813-1.894) | 0.317 | 1.121 (0.734-1.712) | 0.598 | 1.232 (0.807-1.880) | 0.334 | 1.430 (0.930-2.197) | 0.103 |
| N2 | 1.606 (1.120-2.303) | 0.010* | 2.140 (1.456-3.144) | <0.001* | 2.231 (1.529-3.257) | <0.001* | 2.160 (1.480-3.154) | <0.001* | 2.275 (1.557-3.322) | <0.001* | 2.505 (1.714-3.661) | <0.001* |
| N3 | 2.385 (1.660-3.425) | <0.001* | 2.767 (1.893-4.044) | <0.001* | 2.774 (1.911-4.027) | <0.001* | 2.827 (1.950-4.098) | <0.001* | 2.912 (2.001-4.237) | <0.001* | 3.010 (2.074-4.366) | <0.001* |
| Tumor differentiations |  | 0.044* |  | 0.147 |  | 0.410 |  | 0.377 |  | 0.528 |  | 0.385 |
| Poorly | Reference |  | Reference |  | Reference |  | Reference |  | Reference |  | Reference |  |
| Moderately | 0.878 (0.611-1.263) | 0.484 | 0.784 (0.535-1.147) | 0.210 | 0.869 (0.597-1.267) | 0.466 | 0.946 (0.648-1.381) | 0.775 | 0.925 (0.634-1.348) | 0.684 | 0.941 (0.646-1.370) | 0.751 |
| Well | 0.246 (0.079-0.767) | 0.016* | 0.399 (0.126-1.259) | 0.117 | 0.510 (0.162-1.611) | 0.251 | 0.445 (0.141-1.406) | 0.168 | 0.532 (0.168-1.685) | 0.283 | 0.452 (0.144-1.423) | 0.175 |
| Vascular invasion | 1.256 (1.019-1.548) | 0.033* | 1.146 (0.925-1.420) | 0.212 | 1.089 (0.879-1.351) | 0.435 | 1.120 (0.904-1.389) | 0.299 | 1.129 (0.910-1.400) | 0.271 | 1.111 (0.896-1.377) | 0.338 |
| Adjuvant therapy | 0.759 (0.613-0.940) | 0.011* | 0.739 (0.591-0.923) | 0.008* | 0.715 (0.573-0.893) | 0.003* | 0.702 (0.563-0.875) | 0.002* | 0.702 (0.563-0.875) | 0.002* | 0.727 (0.584-0.906) | 0.005* |
| White blood cell (x10^9^/L) | 1.115 (1.037-1.198) | 0.003* | 1.097 (1.019-1.182) | 0.015* | 1.138 (1.059-1.221) | <0.001* | 1.146 (1.068-1.230) | <0.001* | 1.142 (1.064-1.226) | <0.001* | 1.131 (1.054-1.214) | <0.001* |
| Hemoglobin (g/L) | 0.994 (0.990-0.998) | 0.004* | 0.994 (0.990-0.999) | 0.013* | 0.994 (0.989-0.998) | 0.005* | 0.994 (0.990-0.999) | 0.009* | 0.994 (0.990-0.998) | 0.007* | 0.993 (0.989-0.998) | 0.003* |
| Albumin (g/L) | 0.916 (0.895-0.937) | <0.001* | 0.964 (0.940-0.988) | 0.004* | 0.925 (0.903-0.947) | < 0.001* | 0.923 (0.901-0.945) | <0.001* | 0.928 (0.906-0.950) | <0.001* | 0.932 (0.910-0.955) | <0.001* |
| SMI×GNRI |  | <0.001* |  | <0.001* | - | | - | | - | | - | |
| Q1 | Reference |  | Reference |  | - | | - | | - | | - | |
| Q2 | 0.362 (0.280-0.468) | <0.001* | 0.410 (0.314-0.535) | <0.001* | - | | - | | - | | - | |
| Q3 | 0.183 (0.136-0.245) | <0.001* | 0.206 (0.150-0.283) | <0.001* | - | | - | | - | | - | |
| Q4 | 0.123 (0.089-0.172) | <0.001* | 0.140 (0.095-0.204) | <0.001* | - | | - | | - | | - | |
| SATI×GNRI |  | <0.001* | - | |  | 0.002* | - | | - | | - | |
| Q1 | Reference |  | - | | Reference |  | - | | - | | - | |
| Q2 | 0.710 (0.539-0.935) | 0.015* | - | | 0.818 (0.607-1.104) | 0.189 | - | | - | | - | |
| Q3 | 0.504 (0.378-0.671) | <0.001* | - | | 0.596 (0.432-0.823) | 0.002* | - | | - | | - | |
| Q4 | 0.414 (0.310-0.554) | <0.001* | - | | 0.553 (0.395-0.774) | <0.001* | - | | - | | - | |
| VATI×GNRI |  | <0.001* | - | | - | |  | <0.001* | - | | - | |
| Q1 | Reference |  | - | | - | | Reference |  | - | | - | |
| Q2 | 0.603 (0.458-0.794) | <0.001* | - | | - | | 0.632 (0.473-0.844) | 0.002* | - | | - | |
| Q3 | 0.610 (0.462-0.807) | <0.001* | - | | - | | 0.655 (0.485-0.883) | 0.006* | - | | - | |
| Q4 | 0.405 (0.300-0.546) | <0.001* | - | | - | | 0.394 (0.278-0.558) | <0.001* | - | | - | |
| TATI×GNRI |  | <0.001* | - | | - | | - | |  | <0.001* | - | |
| Q1 | Reference |  | - | | - | | - | | Reference |  | - | |
| Q2 | 0.668 (0.509-0.877) | 0.004* | - | | - | | - | | 0.750 (0.562-1.001) | 0.051 | - | |
| Q3 | 0.476 (0.358-0.631) | <0.001* | - | | - | | - | | 0.501 (0.365-0.687) | <0.001* | - | |
| Q4 | 0.375 (0.279-0.504) | <0.001* | - | | - | | - | | 0.419 (0.294-0.597) | <0.001* | - | |
| SMD×GNRI |  | <0.001* | - | | - | | - | | - | |  | <0.001* |
| Q1 | Reference |  | - | | - | | - | | - | | Reference |  |
| Q2 | 0.575 (0.438-0.754) | <0.001* | - | | - | | - | | - | | 0.660 (0.496-0.878) | 0.004* |
| Q3 | 0.507 (0.383-0.671) | <0.001* | - | | - | | - | | - | | 0.643 (0.480-0.862) | 0.003* |
| Q4 | 0.323 (0.239-0.435) | <0.001* | - | | - | | - | | - | | 0.424 (0.308-0.584) | <0.001* |

Supplementary Table 5. Multivariate analysis of the combination of body composition parameters and GNRI and disease-free survival in GC patients.

BMI, body mass index; CEA, carcinoembryonic antigen; GNRI, geriatric nutritional risk index; NRS-2002, nutrition risk screening-2002; SMI, skeletal muscle index; SATI, subcutaneous adipose tissue index; VATI, visceral adipose tissue index; TATI, total adipose tissue index; SMD, skeletal muscle density; HR, hazard ratio; CI, confidence interval.

*P < 0.05 was considered significant.

| Variables | Male | | |  | Female | | |
| --- | --- | --- | --- | --- | --- | --- | --- |
|  | Q1/Q2 | Q2/Q3 | Q3/Q4 |  | Q1/Q2 | Q2/Q3 | Q3/Q4 |
| SMI | 44.3 | 50.2 | 53.7 |  | 39.8 | 41.9 | 49.4 |
| SATI | 25.2 | 36.0 | 50.0 |  | 34.4 | 47.4 | 63.6 |
| VATI | 24.6 | 40.4 | 54.7 |  | 22.1 | 32.8 | 45.2 |
| TATI | 49.5 | 79.8 | 102.9 |  | 63.2 | 85.5 | 103.9 |
| SMD | 33.9 | 39.1 | 44.2 |  | 28.7 | 32.1 | 36.3 |
| SMI×GNRI | 4419 | 4997 | 5511 |  | 3801 | 4181 | 4825 |
| SATI×GNRI | 2484 | 3597 | 4955 |  | 3388 | 4649 | 6284 |
| VATI×GNRI | 2377 | 3993 | 5538 |  | 2164 | 3219 | 4417 |
| TATI×GNRI | 4981 | 7922 | 10399 |  | 6112 | 8293 | 10436 |
| SMD×GNRI | 3403 | 3929 | 4444 |  | 2817 | 3200 | 3647 |

Supplementary Table 6. The sex-specific cutoff values for each body composition parameter and their combinations with GNRI in the training cohort.

SMI, skeletal muscle index; SATI, subcutaneous adipose tissue index; VATI, visceral adipose tissue index; TATI, total adipose tissue index; SMD, skeletal muscle density; GNRI, geriatric nutritional risk index.

| Variables | | Age | | Sex | | BMI | |
| --- | --- | --- | --- | --- | --- | --- | --- |
|  |  | ≤70 | >70 | male | female | < 25 (kg/m2) | ≥ 25 (kg/m2) |
|  |  | HR(95%CI) P value | HR(95%CI) P value | HR(95%CI) P value | HR(95%CI) P value | HR(95%CI) P value | HR(95%CI) P value |
| OS | SMI*GNRI | <0.001* | <0.001* | <0.001* | <0.001* | <0.001* | <0.001* |
|  | Q1 | Reference | Reference | Reference | Reference | Reference | Reference |
|  | Q2 | 0.713(0.477-1.067) 0.100 | 0.359(0.245-0.526) <0.001* | 0.461(0.330-0.644) <0.001* | 0.411(0.247-0.685) <0.001* | 0.398(0.287-0.552) <0.001* | 0.484(0.269-0.872) 0.016* |
|  | Q3 | 0.327(0.204-0.526) <0.001* | 0.137(0.083-0.224) <0.001* | 0.177(0.116-0.269) <0.001* | 0.232(0.127-0.425) <0.001* | 0.178(0.116-0.274) <0.001* | 0.142(0.073-0.278) <0.001* |
|  | Q4 | 0.255(0.151-0.431) <0.001* | 0.093(0.053-0.164) <0.001* | 0.210(0.131-0.334) <0.001* | 0.066(0.031-0.139) <0.001* | 0.122(0.065-0.228) <0.001* | 0.114(0.059-0.220) <0.001* |
|  | ^a^P for trend | <0.001* | <0.001* | <0.001* | <0.001* | <0.001* | <0.001* |
| DFS | SMI*GNRI | <0.001* | <0.001* | <0.001* | <0.001* | <0.001* | <0.001* |
|  | Q1 | Reference | Reference | Reference | Reference | Reference | Reference |
|  | Q2 | 0.658(0.441-0.981) 0.040* | 0.341(0.233-0.498) <0.001* | 0.417(0.298-0.584) <0.001* | 0.415(0.254-0.679) <0.001* | 0.385(0.278-0.533) <0.001 | 0.412(0.231-0.733) 0.003 |
|  | Q3 | 0.329(0.209-0.518) <0.001* | 0.162(0.103-0.256) <0.001* | 0.195(0.131-0.290) <0.001* | 0.304(0.174-0.531) <0.001* | 0.220(0.148-0.327) <0.001 | 0.139(0.074-0.261) <0.001 |
|  | Q4 | 0.244(0.146-0.409) <0.001* | 0.124(0.076-0.202) <0.001* | 0.198(0.126-0.310) <0.001* | 0.100(0.054-0.188) <0.001* | 0.141(0.079-0.254) <0.001 | 0.119(0.065-0.220) <0.001 |
|  | ^a^P for trend | <0.001* | <0.001* | <0.001* | <0.001* | <0.001* | <0.001* |

Supplementary Table 7. Overall survival and disease-free survival of patients with gastric cancer based on quartiles of SMI×GNRI stratified by age, sex, BMI.

The analysis is adjusted for: age, sex, BMI, NRS-2002, adjuvant therapy, pathological T stage, pathological N stage, vascular invasion, tumor differentiation. In each case, the analysis is not adjusted for the stratification variable.

^a^ Test for trend based on variable containing median value for each quintile.

OS, overall survival; DFS, disease-free survival; GNRI, geriatric nutritional risk index; SMI, skeletal muscle index; HR, hazard ratio; CI, confidence interval

*P < 0.05 was considered significant.

| Variables | | NRS-2002 | | Pathological T stage | | Pathological N stage | |
| --- | --- | --- | --- | --- | --- | --- | --- |
|  |  | < 3 | ≥ 3 | T1 and T2 | T3 and T4 | N0 and N1 | N2 and N3 |
|  |  | HR(95%CI) P value | HR(95%CI) P value | HR(95%CI) P value | HR(95%CI) P value | HR(95%CI) P value | HR(95%CI) P value |
| OS | SMI*GNRI | <0.001* | <0.001* | <0.001* | <0.001* | <0.001* | <0.001* |
|  | Q1 | Reference | Reference | Reference | Reference | Reference | Reference |
|  | Q2 | 0.258(0.154-0.431) <0.001* | 0.603(0.436-0.833) 0.002* | 0.364(0.189-0.703) 0.003* | 0.478(0.356-0.642) <0.001* | 0.626(0.362-1.083) 0.094 | 0.403(0.291-0.558) <0.001* |
|  | Q3 | 0.218(0.126-0.376) <0.001* | 0.175(0.112-0.271) <0.001* | 0.245(0.127-0.474) <0.001* | 0.183(0.121-0.278) <0.001* | 0.285(0.150-0.540) <0.001* | 0.181(0.118-0.280) <0.001* |
|  | Q4 | 0.134(0.076-0.238) <0.001* | 0.132(0.077-0.229) <0.001* | 0.229(0.113-0.464) <0.001* | 0.123(0.076-0.198) <0.001* | 0.245(0.123-0.488) <0.001* | 0.121(0.074-0.199) <0.001* |
|  | ^a^P for trend | <0.001* | <0.001* | 0.003* | <0.001* | <0.001* | <0.001* |
| DFS | SMI*GNRI | <0.001* | <0.001* | <0.001* | <0.001* | <0.001* | <0.001* |
|  | Q1 | Reference | Reference | Reference | Reference | Reference | Reference |
|  | Q2 | 0.266(0.159-0.446) <0.001* | 0.538(0.392-0.739) <0.001* | 0.338(0.173-0.660) 0.001* | 0.447(0.334-0.599) <0.001* | 0.505(0.287-0.888) 0.018* | 0.406(0.296-0.556) <0.001* |
|  | Q3 | 0.298(0.178-0.499) <0.001* | 0.177(0.117-0.269) <0.001* | 0.358(0.193-0.663) 0.001* | 0.186(0.126-0.274) <0.001* | 0.365(0.199-0.670) 0.001* | 0.188(0.126-0.281) <0.001* |
|  | Q4 | 0.137(0.078-0.241) <0.001* | 0.178(0.112-0.283) <0.001* | 0.248(0.122-0.504) <0.001* | 0.142(0.093-0.218) <0.001* | 0.270(0.139-0.525) <0.001* | 0.139(0.089-0.217) <0.001* |
|  | ^a^P for trend | <0.001* | <0.001* | 0.004* | <0.001* | <0.001* | <0.001* |

Supplementary Table 8. Overall survival and disease-free survival of patients with gastric cancer based on quartiles of SMI×GNRI stratified by NRS-2002,pathological T stage, pathological N stage.

The analysis is adjusted for: age, sex, BMI, NRS-2002, adjuvant therapy, pathological T stage, pathological N stage, vascular invasion, tumor differentiation. In each case, the analysis is not adjusted for the stratification variable.

^a^ Test for trend based on variable containing median value for each quintile.

OS, overall survival; DFS, disease-free survival; GNRI, geriatric nutritional risk index; SMI, skeletal muscle index; HR, hazard ratio; CI, confidence interval. *P < 0.05 was considered significant.

| Variables | | Tumor differentiations | | Vascular invasion | | Adjuvant therapy | |
| --- | --- | --- | --- | --- | --- | --- | --- |
|  |  | Poorly | Moderately and Well | No | Yes | No | Yes |
|  |  | HR (95% CI) P value | HR (95% CI) P value | HR (95% CI) P value | HR (95% CI) P value | HR (95% CI) P value | HR (95% CI) P value |
| OS | SMI*GNRI | <0.001* | 0.005* | <0.001* | <0.001* | <0.001* | <0.001* |
|  | Q1 | Reference | Reference | Reference | Reference | Reference | Reference |
|  | Q2 | 0.452(0.340-0.600) <0.001* | 0.981(0.301-3.202) 0.975 | 0.491(0.314-0.767) 0.002* | 0.417(0.294-0.592) <0.001* | 0.447(0.282-0.709) <0.001* | 0.439(0.307-0.628) <0.001* |
|  | Q3 | 0.202(0.142-0.287) <0.001* | 0.106(0.021-0.536) 0.007* | 0.220(0.125-0.387) <0.001* | 0.190(0.124-0.293) <0.001* | 0.102(0.049-0.211) <0.001* | 0.297(0.198-0.446) <0.001* |
|  | Q4 | 0.156(0.105-0.230) <0.001* | 0.074(0.014-0.404) 0.003* | 0.145(0.081-0.260) <0.001* | 0.133(0.080-0.221) <0.001* | 0.123(0.063-0.242) <0.001* | 0.173(0.109-0.275) <0.001* |
|  | ^a^P for trend | <0.001* | <0.001* | <0.001* | <0.001* | <0.001* | <0.001* |
| DFS | SMI*GNRI | <0.001* | <0.001* | <0.001* | <0.001* | <0.001* | <0.001* |
|  | Q1 | Reference | Reference | Reference | Reference | Reference | Reference |
|  | Q2 | 0.431(0.325-0.571) <0.001* | 0.519(0.180-1.491) 0.223 | 0.450(0.296-0.684) <0.001* | 0.367(0.256-0.528) <0.001* | 0.385(0.243-0.610) <0.001* | 0.420(0.296-0.598) <0.001* |
|  | Q3 | 0.220(0.158-0.307) <0.001* | 0.205(0.052-0.812) 0.024* | 0.205(0.121-0.346) <0.001* | 0.244(0.162-0.367) <0.001* | 0.126(0.068-0.235) <0.001* | 0.297(0.201-0.440) <0.001* |
|  | Q4 | 0.173(0.120-0.249) <0.001* | 0.065(0.014-0.301) <0.001* | 0.130(0.074-0.228) <0.001* | 0.173(0.110-0.272) <0.001* | 0.125(0.067-0.231) <0.001* | 0.180(0.115-0.280) <0.001* |
|  | ^a^P for trend | <0.001* | <0.001* | <0.001* | <0.001* | <0.001* | <0.001* |

Supplementary Table 9. Overall survival and disease-free survival of patients with gastric cancer based on quartiles of SMI×GNRI stratified by adjuvant therapy, vascular invasion, tumor differentiation.

The analysis is adjusted for: age, sex, BMI, NRS-2002, adjuvant therapy, pathological T stage, pathological N stage, vascular invasion, tumor differentiation. In each case, the analysis is not adjusted for the stratification variable.

^a^ Test for trend based on variable containing median value for each quintile.

OS, overall survival; DFS, disease-free survival; GNRI, geriatric nutritional risk index; SMI, skeletal muscle index; HR, hazard ratio; CI, confidence interval

*P < 0.05 was considered significant.

| Variables | OS | | | DFS | | |
| --- | --- | --- | --- | --- | --- | --- |
|  | AICc | ^a^c-Statistics | *P | AICc | ^a^c-Statistics | *P |
| SMI | 1464.4 | 0.714 | <0.001 | 1547.1 | 0.698 | <0.001 |
| SATI | 1509.3 | 0.638 | <0.001 | 1580.8 | 0.620 | <0.001 |
| VATI | 1524.1 | 0.593 | <0.001 | 1587.6 | 0.585 | <0.001 |
| TATI | 1500.3 | 0.659 | <0.001 | 1576.4 | 0.633 | <0.001 |
| SMD | 1527.1 | 0.609 | <0.001 | 1594.6 | 0.582 | <0.001 |
| SMI×GNRI | 1441.4 | 0.734 | Reference | 1516.6 | 0.723 | Reference |
| SATI×GNRI | 1490.5 | 0.657 | <0.001 | 1565.5 | 0.638 | <0.001 |
| VATI×GNRI | 1518.3 | 0.606 | <0.001 | 1583.8 | 0.603 | <0.001 |
| TATI×GNRI | 1494.3 | 0.665 | <0.001 | 1567.0 | 0.645 | <0.001 |
| SMD×GNRI | 1513.7 | 0.659 | <0.001 | 1581.2 | 0.636 | <0.001 |

Supplementary Table 10. The predictive and discriminatory power of different body composition parameters and the combinations with GNRI in validation cohort.

^a^ The comparison of c-statistics was performed using variance estimation.

OS, overall survival; DFS, disease-free survival; SMI, skeletal muscle index; SATI, subcutaneous adipose tissue index; VATI, visceral adipose tissue index; TATI, total adipose tissue index; SMD, skeletal muscle density; GNRI, geriatric nutritional risk index.

*P < 0.05 was considered significant.
